# NO: a key player in microbiome dynamics and cancer pathogenesis

**DOI:** 10.3389/fcimb.2025.1532255

**Published:** 2025-06-26

**Authors:** Seyedeh Kimia Jasemi, Hossein Faridafshar, Mohammed Namiq Amin, Mehregan Babamohamadi, Marjan Falahati, Roshanak Amirian, Zhila Izadi

**Affiliations:** ^1^ Student Research Committee, Kermanshah University of Medical Sciences, Kermanshah, Iran; ^2^ Universal Scientific Education and Research Network (USERN) Office, Kermanshah University of Medical Sciences, Kermanshah, Iran; ^3^ Department of Microbiology, School of Medicine, Kermanshah University of Medical Sciences, Kermanshah, Iran; ^4^ Department of Microbiology, Faculty of Medicine, Shahed University, Tehran, Iran; ^5^ Department of Biology, School of Natural Sciences, University of Tabriz, Tabriz, Iran; ^6^ Pharmaceutical Sciences Research Center, Health Institute, Kermanshah University of Medical Sciences, Kermanshah, Iran

**Keywords:** nitric oxide, dietary nitrate, nitrite (NONO_2_^-^), nitrate (NO_3_^-^), oral microbiome, microbiota, gastrointestinal cancers, iNOS

## Abstract

The human microbiome refers to the genomic content of microorganisms inhabiting the human body, including the lungs, oral cavity, intestinal tract, esophagus, and other areas. The human oral microbiota is a diverse and complex ecosystem that includes bacteria, microeukaryotes, archaea, and viruses. These communities have a highly structured biogeography resulting from the various microenvironments in the oral cavity, shaping local metabolic exchange. Dietary nitrate (NO_3_^-^) is an ion naturally present in vegetables, especially leafy greens. When consumed, it leads to the production of nitric oxide (NO). This bioactive molecule benefits bodily functions like host defense and neuronal communication and improves vascular and metabolic health. Dietary NO_3_^-^ is reduced to NO via the nitrate-nitrite-NO pathway, facilitated by nitrate-reducing bacteria inside the oral cavity. NO has a leading role in different types of diseases, including cancer, cardiovascular disease, and diabetes. The bioavailability of NO is greatly enhanced by the activity of bacteria residing in the mouth, which reduces NO_3_^-^to NO_2_^-^ and increases the concentration of circulating NO_2_^-^. NO is the key to causing different malignancies, including gastrointestinal cancers. NO can cause cell death by inducing DNA damage and anti-apoptotic signaling pathways. Low to moderate levels of NO derived from tumors can activate angiogenesis and promote an invasive phenotype, while high levels of NO may have an anti-tumor effect in protecting against cancer. In this review, we intend to discuss the human microbiome, dietary NO_3_^-^consumption, the vital role of NO in the human body, types of cancers, and treatments based on it.

## 1 Introduction

The human microbiome refers to the diverse microbial communities and their collective genetic material that inhabit various regions of the human body (Morrison et al., 2023). These microbial residents, encompassing bacteria, fungi, viruses, and archaea, inhabit a variety of anatomical niches, including the lungs, oral cavity, intestinal tract, esophagus, and skin. Each locale nurtures its distinct microbial community, meticulously sculpted by environmental factors such as pH, oxygen levels, and nutrient availability. Far from being passive occupants, these microorganisms are active participants in our physiology, playing critical roles in digestion, immune regulation, and defense against pathogens (Kozak and Pawlik, 2023; Morrison et al., 2023).

The human microbiome occupies specific anatomical niches and significantly impacts essential physiological processes, such as immune regulation, metabolic function, and homeostasis maintenance. The gut microbiota interacts with the host immune system, promoting the development and maturation of immune responses and affecting susceptibility to inflammatory and autoimmune diseases (Belkaid and Hand, 2014). Microbiota-driven metabolism plays a crucial role in nutrient digestion, energy balance, and metabolic signaling pathways, influencing metabolic health and contributing to conditions like obesity and type 2 diabetes (Fan and Pedersen, 2021). Microbiota-derived signals are crucial for maintaining physiological homeostasis, as demonstrated by their role in regulating barrier integrity and local immune tolerance at mucosal surfaces (Thaiss et al., 2016). The functional impact of the microbiome significantly surpasses its compositional diversity across various body sites, highlighting its importance in both health and disease.

The tongue, teeth, gums, and salivary glands each harbor unique microbial populations, engaged in complex metabolic interactions reflective of their specific microenvironments (Arweiler and Netuschil, 2016)., This dynamic ecosystem is shaped by an array of influences, including diet, oral hygiene practices, age, genetics, and overall health status. Beyond its role as a sentinel of oral well-being, the oral microbiome exerts systemic effects, with recent studies implicating it in conditions extending well beyond the mouth, including oncological processes (Schwartz et al., 2021; Wade, 2021).

A remarkable nexus between the oral microbiome and systemic health emerges through the metabolism of dietary nitrates (NO_3_^-^), naturally occurring compounds plentiful in vegetables like spinach, beets, and leafy greens. Upon ingestion, these NO_3_^-^ initiate an elegant biochemical cascade that culminates in the production of NO, a molecule of profound physiological importance. NO serves as a multifaceted signaling agent, renowned for its contributions to vasodilation, neurotransmission, and immune response. Yet, in the realm of cancer, NO exhibits a striking duality: it may either foster tumor progression or suppress malignancy, a behavior contingent upon its concentration, exposure duration, and the surrounding biological context (Hezel and Weitzberg, 2015; Bedale et al., 2016; Bryan et al., 2022; Morou-Bermúdez et al., 2022; Moran et al., 2024; Olas, 2024; Luetic et al., 2025).

**Figure 1:**
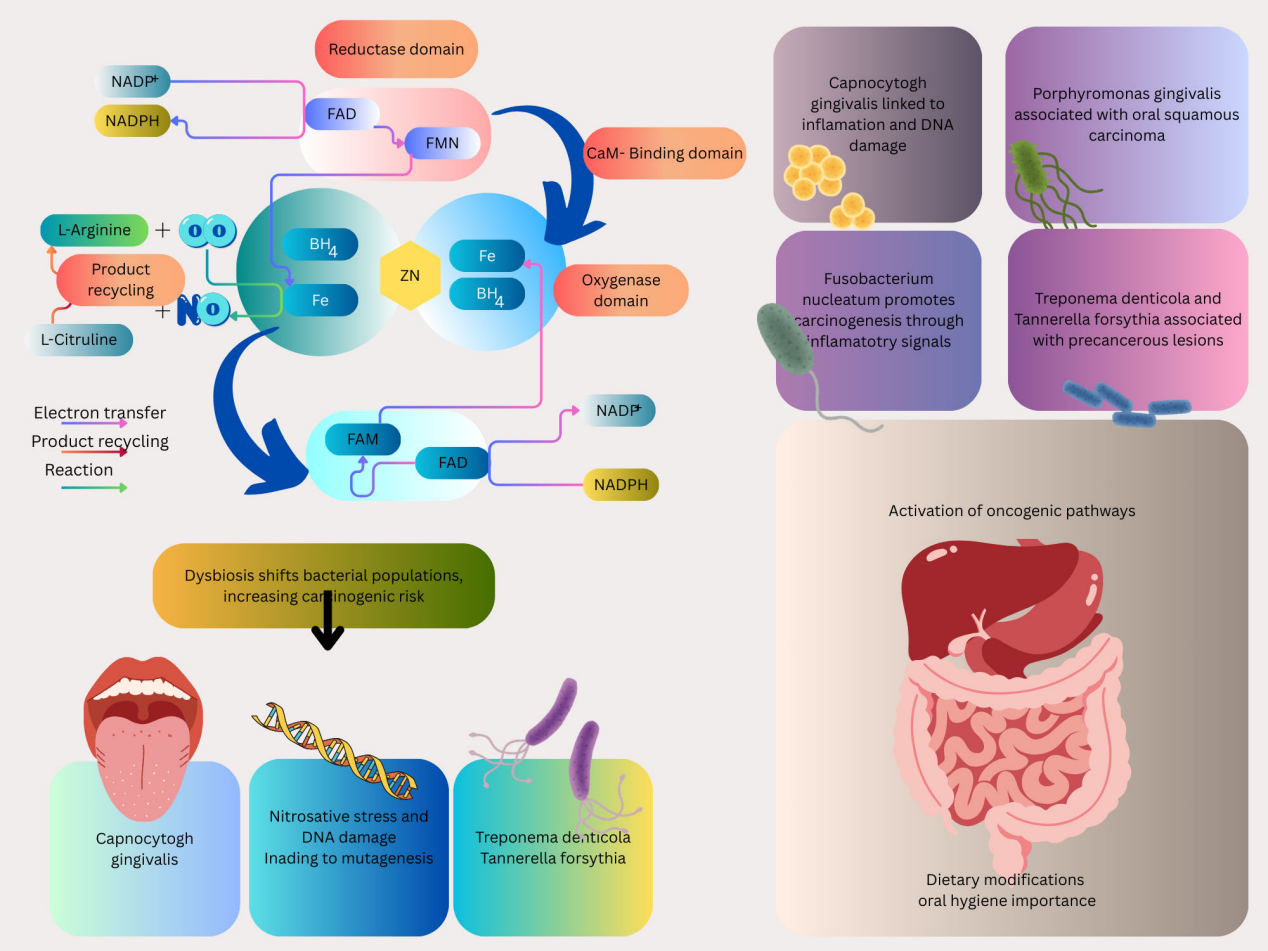
Schematic illustrating the role of dietary NO_3_^-^ in nitrogen cycle and oral microbiota in metabolizing dietary NO_3_^-^ into NO_2_^-^ and NO, highlighting specific bacterial species involved.

Importantly, this microbiome-mediated conversion of dietary NO_3_^-^ to NO_2_^-^ and NO has significant implications for cancer pathogenesis, particularly in the gastrointestinal tract. NO_2_^-^, produced by oral bacteria such as *Veillonella* and *Rothia*, can lead to the formation of carcinogenic nitrosamines in the acidic stomach environment, increasing the risk of gastric cancer (**Figure 1**). Similarly, in esophageal cancer, the reflux of NO_2_^-^rich gastric contents contributes to nitrosative stress and DNA damage in the esophageal epithelium (Lundberg et al., 2008a; Hyde et al., 2014). In colorectal cancer (CRC), NO_2_^-^ from swallowed saliva, along with translocated oral bacteria like *Fusobacterium nucleatum*, can influence the colonic microenvironment, potentially modulating tumor progression through NO-mediated signaling. Even in cancers outside the gastrointestinal tract, such as pancreatic cancer and glioblastoma, systemic NO levels, partly derived from microbiome-mediated NO_3_^-^metabolism, may play a role (Hyde et al., 2014). Thus, the oral microbiome serves as a critical link between dietary NO_3_^-^intake, NO production, and cancer risk, with dysbiosis, a condition known as the repercussions can be significant, potentially altering the abundance or activity of NO_2_^-^producing bacteria and affecting tumorigenesis (Hezel and Weitzberg, 2015; Blekkenhorst et al., 2018; Khan et al., 2020).

NO’s dualistic nature, capable of either promoting or inhibiting tumorigenesis, renders it a focal point in oncological research (Korde Choudhari et al., 2013; Kandalai et al., 2023). At modest concentrations, NO may drive carcinogenesis by enhancing angiogenesis, suppressing programmed cell death (apoptosis), and fostering an invasive tumor phenotype. At elevated levels, NO can inflict DNA damage, exert cytotoxic effects, and mobilize immune-mediated tumor suppression. This behavior positions the oral microbiome, via its modulation of NO synthesis, as a potential influencer of cancer risk and progression, particularly in gastrointestinal malignancies where microbially derived NO exerts its most immediate effects (Kamm et al., 2019; Król and Kepinska, 2020; Mintz et al., 2021). In this exploration, we will undertake a thorough examination of the human microbiome, with a special emphasis on the oral cavity and its pivotal role in mediating the effects of dietary NO_3_^-^ on NO production. We will probe the multifaceted roles of NO in health and disease, with a keen focus on its implications for cancer pathogenesis, particularly within the gastrointestinal tract. Additionally, assess emerging NO-based therapeutic modalities, ranging from NO donors to immunomodulatory agents, and their promise in augmenting existing cancer therapies. By illuminating these intricate relationships, aim to deepen our grasp of cancer biology and catalyze the development of transformative approaches to patient care.

## 2 Dietary nitrate consumption

NO_3_^-^ and NO_2_^-^ are ubiquitous chemical compounds naturally present in a wide array of dietary sources, including vegetables, fruits, processed foods, drinking water, and also serve as food additives in certain products. Vegetables constitute the primary source of dietary NO_3_^-^, accounting for approximately 80–90% of intake in most populations (Hord et al., 2009). Among these, leafy greens such as spinach, arugula, lettuce, Swiss chard, and celery are notably rich, frequently containing NO_3_^-^concentrations exceeding 2500 mg kg^-^¹ fresh weight (Luetic et al., 2025). Root vegetables, including beetroot, and cruciferous vegetables like cabbage and broccoli also contribute substantial amounts, with levels influenced by soil composition, irrigation, and agricultural practices, such as the application of nitrogen-based fertilizers.

Fruits, while less nitrate-dense than vegetables, are noteworthy contributors to dietary intake. Apples, pears, grapes, berries, bananas, melons, and stone fruits typically contain NO_3_^-^ in the range of 10–100 mg kg (Colla et al., 2018; Olas, 2024). Additional sources include grains, pod vegetables, mushrooms, onions, and garlic, which harbor lower but detectable NO_3_^-^levels, which are integral to a balanced diet, and are valued not only for their NO_3_^-^content but also for their provision of essential vitamins, minerals, and antioxidants (Bedale et al., 2016; Ferysiuk and Wójciak, 2020).

NO_2_^-^s, by contrast, are less prevalent in fresh produce but are commonly introduced through processed foods, particularly cured meats such as bacon, sausages, and ham. In these products, sodium or potassium NO_2_^-^ is employed as a preservative to enhance color, flavor, and microbial stability (Ferysiuk and Wójciak, 2020). Certain vegetables, including spinach and celery, also contain naturally occurring NO_2_^-^s, though in smaller quantities (Luetic et al., 2023). Drinking water represents another source of both NO_3_^-^ and NO_2_^-^s, with concentrations varying based on geological factors and agricultural runoff, though regulatory standards aim to limit exposure (Ward et al., 2018).

The dietary intake of NO_3_^-^ and NO_2_^-^ reflects regional and cultural dietary patterns. In Western diets, adults typically consume 50–150 mg of NO_3_^-^ daily, predominantly from vegetables, while NO_2_^-^ intake is lower, ranging from 0.1–10 mg, largely from processed meats and water (Song et al., 2015; Srour et al., 2023). Preparation methods further influence NO_3_^-^content: boiling, for instance, can reduce vegetable NO_3_^-^levels by 20–50%, whereas fresh or minimally processed produce retains higher concentrations (Tiso and Schechter, 2015).

Moreover, additional biological and dietary aspects could influence how NO_3_^-^, NO_2_^-^s, and their associated chemicals affect the body. Some oral bacteria strains have been found to convert NO_3_^-^present in meals to NO_2_^-^ (Tiso and Schechter, 2015). For example, certain species of *Veillonella or Rothia*, located on the back of the tongue, use saliva NO_3_^-^as an energy source and convert it into NO_2_^-^ (Lundberg et al., 2008b).

Researchers discovered that eating foods high in NO_3_^-^ was linked to a lower risk of gastric cancer and that eating foods high in NO_2_^-^ and NDMA was linked to an increased risk of cancer (Song et al., 2015).

When NO_2_^-^ comes into contact with the stomach’s highly acidic secretions, they are transformed into nitrous acid, which interacts with amines to create nitrosamines (Kobayashi et al., 2015). Generally speaking, dietary NO_2_^-^, the primary nitrosating agent acquired from meals and reduction of salivary NO_3_^-^, is catalyzed in the acidic stomach to form NO-related compounds, such as S-nitroso, N-nitroso, O-nitroso compounds, and NO (Gago et al., 2008; Pinheiro et al., 2015). According to reports, nitrosamines can cause esophageal, gastric, and colon cancers (Park et al., 2015; Bedale et al., 2016; Alaei et al., 2021).

Eating foods rich in NO_3_^-^ and NO_2_^-^s, particularly animal sources, has been linked to an increased risk of cancer, including breast, gastric, renal cell, adult glioma, colorectal, esophageal, and thyroid cancers. Such foods contain high levels of amines and amides, as well as heme iron, which may promote the production of endogenous N-nitroso compounds, thus contributing to cancer risk (Karwowska and Kononiuk, 2020). Also, some data point to a possible connection between CRC and NO_3_^-^ in drinking water (Schullehner et al., 2018; Temkin et al., 2019). This is due to the conversion of NO_3_^-^ into carcinogenic N-nitroso compounds (Schullehner et al., 2018). Thus, eating vegetables(consumption of NO_3_^-^) is seen as a component of a healthy diet for people, whereas increased consumption of NO_2_^-^ and NDMA seems to be a risk factor for cancer (Song et al., 2015).

## 3 Nitric oxide production pathway

NO production begins with the substrate L-arginine, which is converted to NO and L-citrulline by the enzyme NO synthase (NOS). This reaction occurs within different cells, such as endothelial cells, neurons, and macrophages (**Figure 1**). The enzyme NOS exists in three isoforms: endothelial (eNOS), neuronal (nNOS), and inducible (iNOS). Each of these isoforms is involved in a physiological process, including vascular homeostasis, neurotransmission, and immunological defense mechanisms.

Further, the NO produced via this pathway exerts its effects by stimulating guanylate cyclase, leading to increased levels of cyclic guanosine monophosphate (cGMP). This molecule acts as a secondary messenger, resulting in various physiological responses such as vasodilation and inhibition of platelet aggregation. Thus, the L-arginine-NO pathway plays a vital role in maintaining physiological homeostasis (Gonzalez et al., 2025). Endothelial cells, which are critical for NO production through endothelial eNOS, do not play a direct role in the oxidation of NO_2_^-^ to NO_3_^-^under physiological conditions. eNOS catalyzes the conversion of L-arginine to NO and L-citrulline, and the NO produced can be oxidized to NO_2_^-^ in plasma or further to NO_3_^-^by oxyhemoglobin (HbO_2_) in erythrocytes (Ignarro, 1990). However, there is no evidence that endothelial cells themselves possess the enzymatic machinery to oxidize NO_2_^-^ to NO_3_^-^. Instead, endothelial cells may contribute indirectly to NO_2_^-^ dynamics by generating NO, which is then metabolized in the bloodstream to form NO_2_^-^ and NO_3_^-^.

In certain contexts, such as hypoxia, endothelial cells can reduce NO_2_^-^ to NO, mediated by enzymes like xanthine oxidoreductase or eNOS acting as a NO_2_^-^ reductase, enhancing local NO bioavailability (Su et al., 2020). Under inflammatory conditions, endothelial cells may express enzymes like myeloperoxidase, which could theoretically oxidize NO_2_^-^, but this is not a primary function and occurs mainly in pathological states (Pacher et al., 2007). Thus, while endothelial cells are integral to NO synthesis and NO_2_^-^ reduction, their role in NO_2_^-^ oxidation is negligible, with HbO_2_ in the blood serving as the dominant mediator.

This distinction is relevant to cancer biology, as the balance between NO_2_^-^ and NO_3_^-^levels influences the availability of substrates for the NO_3_^-^NO_2_^-^NO pathway, potentially affecting tumor microenvironments. For instance, excessive NO_2_^-^ accumulation due to dysregulated metabolism could contribute to nitrosative stress, promoting DNA damage in gastrointestinal cancers (Lundberg et al., 2008a).

### 3.1 Nitric oxide generation from dietary nitrate

The generation of NO from dietary NO_3_^-^ represents a critical metabolic pathway mediated by the oral microbiome and gastrointestinal environment. Dietary NO_3_^-^, from vegetables, is ingested and absorbed into the bloodstream (Keller et al., 2020; Liu et al., 2020), where they are concentrated in saliva by the salivary glands (**Figure 1**). In the oral cavity, facultative anaerobic bacteria, notably *Veillonella* and *Rothia* species, reduce NO_3_^-^ to NO_2_^-^ through enzymatic activity. These bacteria, residing predominantly on the tongue, utilize NO_3_^-^as an electron acceptor, producing NO_2_^-^ as a metabolic byproduct, as NO, and other reactive nitrogen oxides. NO_2_^-^ can also be converted into NO and other bioactive nitrogen oxides in the blood and tissues by enzymatic and nonenzymatic mechanisms. Interestingly, these mechanisms are accelerated under hypoxic and acidic conditions when oxygen-dependent NOS enzymes may not function properly. Therefore, the NO_3_^-^NO_2_^-^NO pathway is considered a backup system that ensures NO bioactivity when there is low NOS output (Lundberg and Weitzberg, 2022).

Following its production, NO is a highly reactive molecule that undergoes rapid metabolism in the body, with one of its primary fates being oxidation to NO_3_^-^. This process is predominantly mediated by oxyheme-containing proteins, such as (HbO_2_ in red blood cells and oxymyoglobin in muscle tissues. These proteins, which contain heme groups with iron in the ferrous (Fe²^+^) state bound to oxygen, facilitate the oxidation of NO to NO_3_^-^through a series of biochemical reactions (Ignarro, 1990).

In the bloodstream, HbO_2_, is the primary mediator of NO oxidation. NO reacts with oxyhemoglobin to form methemoglobin (where the heme iron is oxidized to Fe³^+^) and NO_3_^-^, as described by the reaction:


$$NO+HbO_{2}\to metHb+NO_{3}^{-}$$

This reaction occurs rapidly in the vascular compartment, particularly in erythrocytes, ensuring that NO is efficiently converted to NO_3_^-^, which is then excreted in urine or recycled through the NO_3_^-^NO_2_^-^NO pathway. The high concentration of HbO_2_ in blood (approximately 10 mM heme) makes this a dominant pathway for NO clearance in the systemic circulation, preventing excessive NO accumulation that could lead to cytotoxicity or vasodilation (Ignarro, 1990).

In tissues, particularly skeletal and cardiac muscle, HbO_2_ plays a similar role. NO reacts with HbO_2_ to produce NO_3_^-^and metmyoglobin, effectively regulating NO levels in these oxygen-rich environments. This process is especially relevant in tissues with high metabolic activity, where NO produced by endothelial or inducible iNOS must be tightly controlled to maintain homeostasis (Shiva, 2013).

Additionally, NO can be oxidized to NO_3_^-^ in other oxygen-rich environments, such as the lungs, where inhaled oxygen or Reactive oxygen species (ROS) may contribute to non-enzymatic oxidation. However, the oxyheme-mediated pathway remains the primary mechanism due to its efficiency and prevalence (Lundberg et al., 2008a). The resulting NO_3_^-^is either excreted by the kidneys or concentrated in saliva by the salivary glands, re-entering the NO_3_^-^NO_2_^-^NO pathway, thus completing a cyclical process that integrates dietary NO_3_^-^ metabolism with endogenous NO turnover.

This oxidation process has implications for cancer biology, as NO_3_^-^ levels in the body, whether derived from dietary sources or NO metabolism, can influence the availability of substrates for the NO_3_^-^NO_2_^-^NO pathway, potentially modulating tumor microenvironment dynamics. For instance, excessive NO_3_^-^production from NO oxidation in dysbiotic conditions could amplify nitrosative stress, contributing to DNA damage and carcinogenesis, particularly in gastrointestinal cancers (Khan et al., 2020).

## 4 Characteristics of the oral microbiota in carcinogenesis

**Figure 2:**
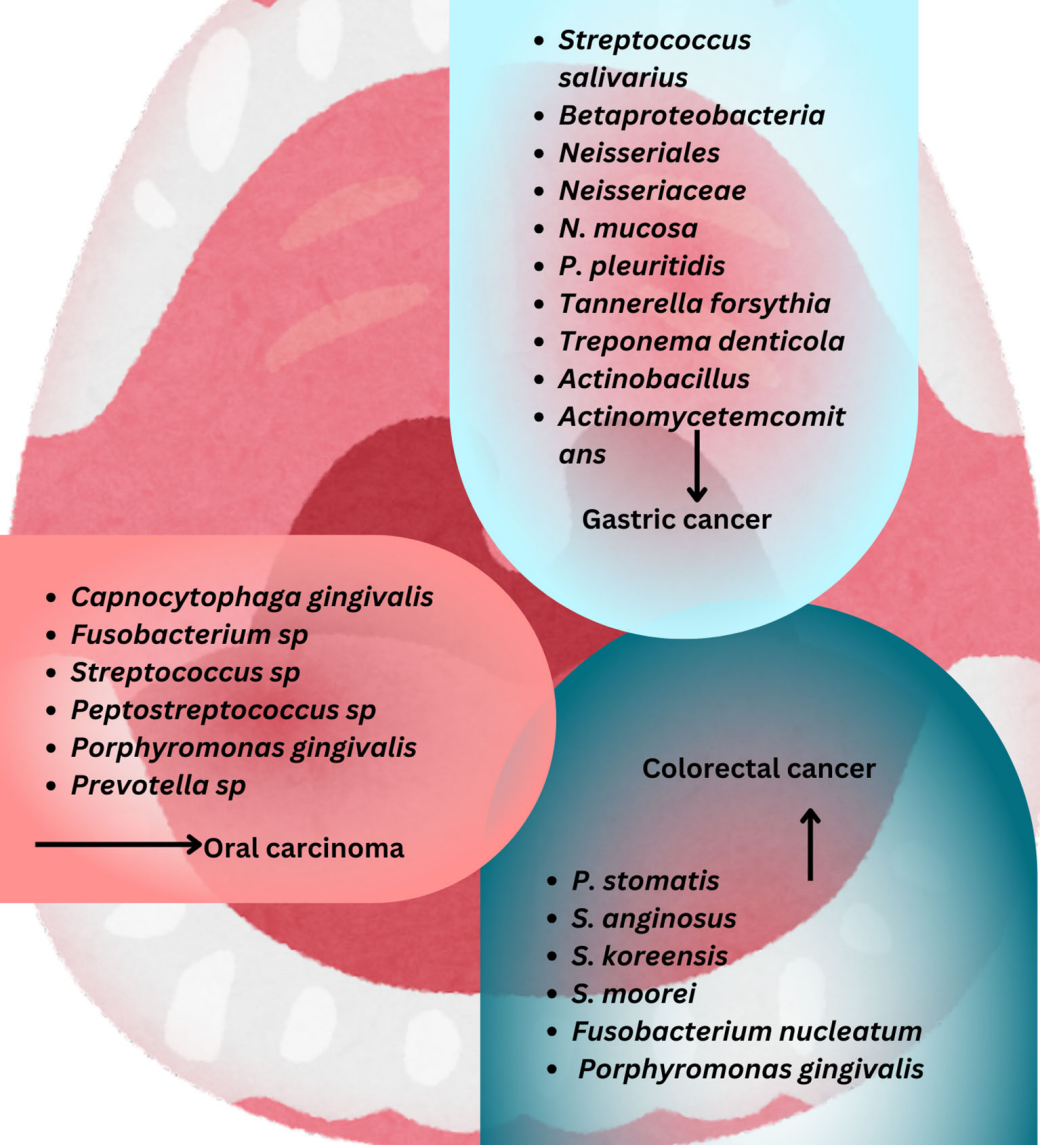
Various cancers-associated bacteria are depicted, which intricate interplay between these microorganisms and NO signaling pathways may affect cancer development and progression.

The oral microbiota, a complex community of over 700 microbial species including bacteria, fungi, viruses, and protozoa, plays a critical role in metabolizing dietary NO_3_^-^ into NO, as outlined in the preceding section (Caselli et al., 2020; Tuominen and Rautava, 2021), is shaped by various factors, such as dietary habits, oral hygiene practices, the host’s immune response, systemic health conditions, and the use of medications. The composition of microbial communities is significantly influenced by diet, which affects nutrient availability and pH levels (Wade, 2013). The immune system, especially innate immunity and mucosal defenses, regulates microbial balance and prevents the overgrowth of pathogenic species (Belkaid and Hand, 2014). Systemic diseases, including diabetes and autoimmune disorders, can disturb microbial balance and lead to oral dysbiosis (Lamont and Hajishengallis, 2015). Furthermore, antimicrobial drug treatments, particularly broad-spectrum antibiotics, may diminish microbial diversity and facilitate the growth of opportunistic pathogens (Bhalodi et al., 2019). Age and hormonal changes influence the composition of oral microbiota over time (Huttenhower et al., 2012; Lloyd-Price et al., 2016). Beyond its contribution to NO production via the NO_3_^-^NO_2_^-^NO pathway, the oral microbiota is increasingly recognized for its role in carcinogenesis, particularly in oral, esophageal, gastric, and colorectal cancers, where dysbiosis may amplify NO-related oncogenic processes (**Figure 2**) (Lundberg et al., 2008a; Pennisi et al., 2017; Zhang et al., 2018; Cali et al., 2019; Sun et al., 2020). Dysbiosis of the oral microbiota can significantly affect the host’s metabolism and immune system and lead to local and systemic disorders such as systemic lupus erythematosus and cancers such as squamous cell carcinoma (SCC) which, in this situation, excessive NO production can lead to nitrosative stress, causing DNA damage and tumor initiation. This mechanism is particularly relevant in esophageal cancer, which affects 604,100 individuals annually (Lamont and Hajishengallis, 2015; Helmink et al., 2019; Li et al., 2020; Sarkar et al., 2021). For instance, one of the risk factors for oral squamous cell carcinomas (OSCC), a subgroup of head and neck (SCC) (Tandon et al., 2017; Bray et al., 2018) is homeostasis dysregulation. Due to the possibility that bacteria may encourage inflammation, cell proliferation, and the production of some oncogenic substances, several species, including *Capnocytophaga gingivalis, Fusobacterium* sp*, Streptococcus* sp*, Peptostreptococcus* sp*, Porphyromonas gingivalis, and Prevotella* sp, have been strongly correlated with oral carcinoma (Karpiński, 2019). *Porphyromonas gingivalis*, *Fusobacterium nucleatum, Treponema denticola, and Streptococcus anginosus*, four common oral cavity residents, have been identified as probable causative agents of OSCC (Zhang et al., 2019).

The findings show that six families (*Prevotellaceae, Fusobacteriaceae, Flavobacteriaceae, Lachnospiraceae, Peptostreptococcaceae*, and *Campylobacteraceae)* and 13 genera, including *Porphyromonas, Alloprevotella, and Fusobacterium*, are enriched in cancer tissues. Also, *Fusobacterium nucleatum, Prevotella intermedia, Aggregatibacter segnis, Capnocytophaga leadbetteri, Peptostreptococcus stomatis*, and five additional species have significantly higher abundances at the species level, indicating a probable link between these bacteria and OSCC (Zhang et al., 2019).

Esophageal adenocarcinoma (EAC) and esophageal squamous cell carcinoma (ESCC) are the two primary histological forms of esophageal cancer (**Figure 2**) (Kelly, 2019) (Uhlenhopp et al., 2020; Sung et al., 2021).

*Fusobacterium nucleatum* has been demonstrated to promote CRC in experimental tests by producing chemokines and activating the E-cadherin/β-catenin signaling pathway via its FadA adhesin (**Figure 2**) (Rubinstein et al., 2013; Franzosa et al., 2014; Ferlay et al., 2015; Derakhshankhah et al., 2017; Douaiher et al., 2017; Siegel et al., 2020; Uchino et al., 2021) (Pignatelli et al., 2021) (Sun et al., 2017). IL-8 and growth-related oncogene (GRO), two CXC-chemokine genes, are expressed in cultured epithelial cells by viable *Streptococcus anginosus* isolated from esophageal cancer tissues (GRO) (Narikiyo et al., 2004). Also, the presence of *Streptococcus anginosus* has been documented in tissues from esophageal cancer, gastric cancer, dysplasia of the esophagus, and head and neck squamous cell carcinomas. These indicate that *Streptococcus anginosus* is involved in the carcinogenic process (Sasaki et al., 1998; Tateda et al., 2000).

Besides oral cancer correlated with oral cavity microbiome, the fourth most prevalent cancer in the world, and the fifth most frequently diagnosed malignancy in gastric cancer, which is associated with oral cavity microbiome, has been correlated with poor oral health (Ndegwa et al., 2018; Sung et al., 2021). The development of gastric cancer and the oral microbiome have been linked. *Streptococcus salivarius* was the first bacterium found in gastric tumors (Ijyuuin and Umehara, 2012). Based on the studies conducted about the oral microbiome and gastric cancer, it has been found that the microbial abundance of *Tenericutes*, M. orale, *E. yurii*, and *Cutibacterium* decreases. In contrast, the abundance of *Betaproteobacteria, Neisseriales, Neisseriaceae, N. mucosa*, and *P. pleuritidis* increases (Yang et al., 2022). In a recent study conducted in the United States, the prevalence of periodontal pathogens was compared in samples of dental plaque and saliva from 35 patients with precancerous gastric lesions and 70 controls. They discovered precancerous gastric lesions were more likely to develop when *Tannerella forsythia*, *Treponema denticola*, and *Actinobacillus actinomycetemcomitans* loads were present (Sun et al., 2017).

Maintaining oral microbial balance through rigorous hygiene practices, such as regular brushing and tongue cleaning, may reduce pathogenic bacterial loads and modulate NO production, potentially lowering cancer risk (Tanda et al., 2019). The oral microbiota’s dual role in NO generation and dysbiosis underscores its significance in carcinogenesis, particularly in the context of dietary NO_3_^-^metabolism, and highlights the need for further research into its mechanistic contributions to cancer development.

## 5 Resilience and composition of oral microbiome

In the context of oral cancer and NO production, resilience refers to the microbiome’s capacity to maintain or restore its compositional and functional stability, particularly its role in metabolizing dietary NO_3_^-^ into NO despite perturbations such as dysbiosis, cancer development, or therapeutic intervention (Krasse, 2001; Trombelli et al., 2004; Izadi et al., 2016; Kilian et al., 2016; Rosier et al., 2018; Zhang et al., 2018; Dashper et al., 2019; Caselli et al., 2020; Shouval et al., 2020; Welch et al., 2020; Ingham et al., 2021; Wade, 2021).

### 5.1 Factors influencing resilience

Resilience is challenged by both cancer development and its treatments. Oral hygiene practices (e.g., brushing, tongue cleaning) and dietary NO_3_^-^intake (e.g., from spinach, beetroot) promote resilience by supporting beneficial bacteria (Blekkenhorst et al., 2018). Conversely, poor hygiene or low NO_3_^-^intake may exacerbate dysbiosis, increasing cancer risk through altered NO dynamics (Rosier et al., 2018). The oral microbiome’s resilience is crucial for maintaining balanced NO production, influencing cancer risk through NO_3_^-^metabolism. Dysbiosis, treatment-induced shifts, and lifestyle factors can disrupt this balance, highlighting the need for strategies like targeted hygiene or dietary interventions to enhance resilience and mitigate oncogenic NO effects.

Chemotherapy and radiotherapy, common in OSCC management, induce significant microbial shifts, reducing beneficial nitrate-reducing bacteria and increasing opportunistic pathogens like *Candida* species (Alnuaimi et al., 2015). These treatments disrupt microbial biofilms, impair salivary function, and exacerbate inflammation, further compromising microbiome resilience (Gaetti-Jardim et al., 2009). For instance, post-treatment reductions in *Veillonella* may diminish NO production from dietary NO_3_^-^, while dysbiotic enrichment of *Fusobacterium nucleatum* may enhance inflammation-driven carcinogenesis via NO-mediated pathways (Gaetti-Jardim et al., 2009). Despite these perturbations, the oral microbiome often demonstrates partial recovery, with resilient taxa like *Streptococcus* and *Actinomyces* recolonizing within months post-treatment, potentially restoring NO_3_^-^metabolism (He et al., 2015).

The interplay between microbiome resilience, oral cancer, and NO production underscores the importance of maintaining microbial balance. Strategies to enhance resilience, such as targeted oral hygiene and dietary interventions, may mitigate dysbiosis and modulate NO-related oncogenic processes, offering potential avenues for cancer prevention and treatment optimization.

## 6 Exploring the role of NO in cancers

NO can lead to mutations primarily through the induction of nitrosative stress. Under conditions where ROS are present, NO rapidly reacts with superoxide anions (O_2_^·−^) to generate PeroxyNO_2_^-^ (ONOO^−^).

ONOO− is a highly reactive oxidant that can modify critical cellular components, including DNA (Radi, 2013). ONOO− and other RNS can cause direct modifications to DNA bases, and nitration and nitrosation reactions may transform guanine into 8-nitroguanine or other altered bases (Pacher et al., 2007; Semenikhina et al., 2022). These modifications can lead to mispairing during DNA replication and eventually result in point mutations. The oxidative and nitrosative stress generated by ONOO− can induce both single-strand and double-strand breaks in DNA. Such strand breaks compromise the integrity of the genome and increase the likelihood of mutagenesis if not adequately repaired (Semenikhina et al., 2022).

Also, NO and its derivatives can impair the function of key DNA repair enzymes. This interference can diminish the cell’s ability to rectify errors arising during DNA replication, allowing mutations to accumulate over time.

Another function in which NO can also contribute to the formation of nitrosamines, especially in acidic environments, which are known to be mutagenic. Although this mechanism is more prominent in the context of dietary NO_2_^-^ and processed meats, it further supports the link between NO-related biochemical reactions and DNA mutation processes (Pacher et al., 2007; Semenikhina et al., 2022).

At low to moderate levels, NO produced by the oral microbiome supports cellular homeostasis and immune surveillance. For instance, in the oral cavity, NO derived from nitrate-reducing bacteria enhances antimicrobial defenses, potentially inhibiting early tumor initiation (Wink et al., 2011; Khan et al., 2020). However, in the context of microbial dysbiosis, excessive NO production can drive oncogenic processes. Dysbiosis, characterized by enrichment of pathogenic bacteria like *Porphyromonas gingivalis* and *Fusobacterium nucleatum* in OSCC, disrupts the balance of nitrate-reducing bacteria, leading to elevated NO levels that induce nitrosative stress (Karpiński, 2019; Sun et al., 2020). This stress causes DNA damage, protein nitrosylation, and activation of oncogenic signaling pathways, promoting tumor initiation and progression in OSCC (Bray et al., 2018).

Similarly, ROS, such as hydrogen peroxide (H_2_O_2_), produced by dysbiotic oral bacteria via enzymes like NADPH oxidase, acts as a second messenger in signaling pathways, promoting cell proliferation at low concentrations or inducing cell death at higher levels. The interplay between NO and ROS amplifies oxidative and nitrosative stress, exacerbating carcinogenesis in OSCC (McBee et al., 2017; Kandalai et al., 2023).

This interplay between the oral microbiome, NO production, and cancer underscores the need for targeted interventions. Strategies to modulate microbial composition, such as probiotics or nitrate-rich diets, may optimize NO levels to favor antitumor effects while minimizing oncogenic nitrosative stress. These insights highlight the oral microbiome’s critical role in shaping NO’s impact on cancer, particularly in the context of dietary NO_3_^-^metabolism. Further illustrates the oral-gut microbiome axis. Oral bacteria such as *Fusobacterium nucleatum* and *Peptostreptococcus stomatis* translocate to the gut via saliva, where they promote NO-driven inflammation and ROS production, enhancing tumor growth (Uchino et al., 2021). *Fusobacterium nucleatum* activates β-catenin signaling, while gut bacteria produce ROS/RNS via NADPH oxidase, contributing to oncogenic signaling in early CRC and potentially tumor-suppressive effects in advanced stages (Ndegwa et al., 2018). The dual role of NO and ROS/RNS highlights their context-dependent effects, with microbiome-driven production shaping cancer outcomes (**Table 1**).

**Table 1:** The results of preclinical studies regarding the different effects of nitric oxide in various types of cancer.

Cancers Types	Study Model	Results	References
Gastric cancer	*In vitro*	The elevated amount of NO was shown in this research to be a potential therapeutic target for gastric cancer.	(Zhang et al., 2018)
Gastric cancer	*In vivo*	Through several established cancer-related processes, increased NOS3 expression promoted the emergence and progression of STAD. Drugs that suppress NOS3 were found, according to drug response analyses.	(Zou et al., 2021)
Pancreatic cancer	*In vitro*	NO controlled release showed dose-dependent cytotoxicity *in vitro*. These data imply that NO is an effective chemosensitizer. A new class of therapies that uses locally regulated release of NO for chemosensitization may change the treatment of pancreatic cancer and other solid tumors with chemoresistance.	(Araujo-Gutierrez et al., 2019)
Esophageal cancer	*In vitro*	According to the study’s findings, treatment with various NO concentrations and/or radiotherapy dosages had an impact on the esophageal cancer cell line TE-1 by increasing cell death and reducing cell growth.	(Sun et al., 2013)
Liver cancer	*In vivo*	Specifically, in a subgroup of individuals with diminished liver Protein arginine methyltransferase 1 (PRMT1) and high iNOS activity levels, the findings provided in this research show that selective iNOS inhibition is a potential approach for treating alcoholic liver disease ALD and alcohol-associated HCC.	(Zhao et al., 2020)
Cholangiocarcinoma	*In vivo*	The bile duct epithelia and hepatocyte cells in the experimental cholangiocarcinoma group showed positive staining for eNOS in their cytoplasm. The untreated group’s bile duct epithelial cells just faintly stained positive for eNOS.	(Suksawat et al., 2017)
Gastric cancer	*In vitro*	The presence of an elevated level of NO for those with gastric cancer has an inhibitory effect on the growing number of cancer cells.	(Yao et al., 2015)
Colon cancer	*In vivo*	The proliferation of cancer cells and a reduction in apoptosis among these cancer cells were seen in an animal colon cancer investigation, despite an elevated NO.	(Monteiro et al., 2015)
Pancreatic ductal adenocarcinoma	*In vivo*	NO levels, as well as the proliferation of cancer cells, were shown to be reduced in an animal study with the pancreatic cancer model.	(Wang et al., 2016a)
Glioblastoma	*In vitro* *In vivo*	NO administration affects GSCs by slowing their growth and enhancing their susceptibility to chemotherapy, which could have implications for long-term management. BET inhibitor targeting of iNOS expression in cancer cells. Such targeting can markedly improve therapeutic efficacy in glioblastoma Targeting the nitrosative stress with HU-53 and HU-54 may be a promising therapeutic strategy for glioblastoma treatment	(Salvatori et al., 2023) (Fahey et al., 2018) (Tripathi et al., 2023)

### 6.1 NO’s role in promoting tumorigenesis

Angiogenesis is a primary pro-tumorigenic effect, as NO stimulates vascular endothelial growth factor (VEGF) expression, promoting blood vessel formation to supply nutrients and oxygen to tumors (Korde Choudhari et al., 2013). This is evident in colorectal and gastric cancers, where NO enhances tumor vascularization, supporting growth and metastasis. Inhibition of apoptosis is another key mechanism, with NO suppressing caspase activity and upregulating anti-apoptotic proteins like Bcl-2, allowing tumor cells to evade programmed cell death (Khan et al., 2020). Additionally, NO facilitates tumor invasion by activating matrix metalloproteinases (MMPs), which degrade extracellular matrix, enabling cancer cell migration, as seen in pancreatic and esophageal cancers (Monteiro et al., 2019). These effects are amplified in dysbiotic conditions, where oral bacteria like *Fusobacterium nucleatum* increase NO production, contributing to tumor progression (Sun et al., 2020). Collectively, these mechanisms highlight NO’s role in fostering an oncogenic environment at low concentrations.

### 6.2 NO’s anti-cancer properties

At high concentrations, NO exhibits anti-cancer properties, counteracting tumor growth through cytotoxic and immune-mediated mechanisms. DNA damage is a primary effect, as elevated NO levels, often from iNOS, induce nitrosative stress, causing single- and double-strand DNA breaks that trigger tumor cell apoptosis (Pacher et al., 2007). This is observed in glioblastoma, where high NO levels sensitize tumor cells to chemotherapy (Tripathi et al., 2023). Immune activation is another critical mechanism, with NO enhancing cytotoxic T-cell and macrophage activity, promoting tumor cell killing. In the oral cavity, NO from nitrate-reducing bacteria supports immune surveillance, potentially inhibiting early tumor initiation. Additionally, high NO levels disrupt mitochondrial function, inducing cytotoxicity and apoptosis in tumor cells, as seen in pancreatic and hepatocellular carcinomas. These anti-tumorigenic effects underscore NO’s potential as a therapeutic agent, particularly when modulated to achieve high local concentrations (Mintz et al., 2021).

### 6.3 Gastrointestinal cancer

GI cancers represent more than one-fourth of all cancer incidence and one-third of cancer-related mortality (Huang et al., 2023). The positive expression of P53, iNOS, and vascular endothelial growth factor is significantly increased in gastric cancer. NOS can induce DNA mutations in tumor suppressor genes, leading to the occurrence of gastric cancer. In gastric cancer, for example, NO can inhibit the growth and proliferation of cancer cells and promote apoptosis in cancer cells. Additionally, NO can also help to regulate inflammation and oxidative stress in the stomach, which can contribute to the development of gastric cancer (Sang et al., 2011; de Oliveira et al., 2017).

iNOS was reported to be involved in the occurrence, development, invasiveness, and metastasis of esophageal carcinoma. Also, high iNOS, eNOS, and NOS mRNA expression has been found in human colorectal cancer tissues. The accumulation and phosphorylation of p53 caused by NO leads to cell cycle arrest, and the levels of iNOS and p53-p-Ser15 positively correlate with the degree of inflammation (Hu et al., 2020).

In esophageal cancer, chronic exposure to NO can lead to the formation of nitrosamines, which are potent carcinogens that can contribute to the development of cancer. However, NO can also help to protect against the development of esophageal cancer by inhibiting the growth and proliferation of cancer cells (Hofseth et al., 2003).

In CRC, NO can have both pro- and anti-cancer effects. In some cases, NO can inhibit the growth and proliferation of cancer cells in the intestines, while in other cases, NO can promote the survival and growth of cancer cells (Wang et al., 2020).

Additionally, NO can also modulate the immune response in the intestines, which can impact the development and progression of cancer (Mokry et al., 2020).

### 6.4 Colorectal cancer

Some microbiomes, including E. Coli and Species belonging to genera Bacteroides and *Prevotella*, have also been noted as being significantly more abundant in CRC patients. The commensal bacteria *Fusobacterium nucleatum* was also found to increase intestinal tumorigenesis without aggravating colitis or inflammatory pathways (Kandalai et al., 2023).

ROS plays an essential role in our bodies as a moderate level of ROS leads to cell damage and promotes cancer; however, an excessively high level of ROS induces cancer cell death, acting as an anti-cancer. ROS are usually higher in CRC cells and induce cell death in cancer cells while not affecting normal cells (Lin et al., 2018). Different NOS3 and NOS1 polymorphisms described in colorectal tissue are suspiciously associated with the development of colon cancer. NOS2 and NOS3 promote colon cancer’s migration and invasive capacity by activating soluble guanylate cyclase (Monteiro et al., 2019).

NO produced by NOS2 exacerbates colon cancer at inflammatory sites. NOS2 is expressed in 50–60% of colon cancer patients, and those with high NOS2 expression have the prognosis. In healthy individuals, NOS2 expression is absent in colon epithelial cells, and it is low-to-intermediate in patients with chronic colitis and is high in patients with colon cancer. In the primary stages of colon cancer, low NOS2 expression is found (Monteiro et al., 2019).

In the case of CRC, *Veillonella* and *Rothia* facilitate the reduction of dietary NO_3_^-^ into NO_2_^-^, which can form carcinogenic nitrosamines in the stomach’s acidic environment, elevating gastric cancer risk. In esophageal cancer, particularly adenocarcinoma, NO_2_^-^ rich saliva from oral microbial activity can reflux into the esophagus, contributing to nitrosative stress and DNA damage (**Table 2**) (Hyde et al., 2014; Camañes-Gonzalvo et al., 2024).

**Table 2:** The results of clinical studies regarding the different effects of nitric oxide in different types of cancer.

Cancers Types	Study Model	Results	References
Gallbladder cancer	Clinical trial	In healthy gallbladder epithelial cells, iNOS expression was not present, however, stromal cells sometimes expressed it positively. In the cytoplasm of the inflammatory cells surrounding the tumor cells in gallbladder carcinoma tissue, iNOS was mostly expressed.	(Niu et al., 2004)
Esophageal cancer	Clinical trial	In comparison to the group that acted as the control, the patients’ levels of NO were found to be significantly higher.	(Sayır et al., 2019)
Esophageal cancer	Clinical trial	There was an increase in eNO levels, but compared to baseline, there was only a slight increase after radiation, and by the time of the follow-up appointment, it had almost completely subsided.	(McCurdy et al., 2011)
Esophageal cancer	*In vitro* & clinical trial	Studies showed that a high luminal NO concentration creates a special environment for Barrett’s oesophagus to form in esophageal stromal cells during esophageal wound healing.	(Sun et al., 2013)
Gastric cancer	Clinical trial	With the development of the tumor, the expression of the cytoprotective enzymes ecNOS and COX-1 in gastric cancer decreased. When compared to less advanced tumors, advanced tumors greater than 5 cm, penetrating into or through the serosa, and having metastases to lymph nodes or distant organs exhibited considerably higher iNOS and COX-2 expression.	(Rajnakova et al., 2001)
Colon cancer	*In vitro* & clinical trial	According to the study, individuals who expressed more NOS1 tended to have shorter overall survival times than those who expressed less NOS1 among all colon cancer patients.	(Wang et al., 2019)
Colon cancer	*In vitro* & clinical trial	According to the current research, human colon cancer feed arteries have improved vasorelaxant and decreased vasoconstrictive function when compared to corresponding normal colon arteries, indicating that they are specialized towards lower resistance. The increased NO-dependent signaling that underlies these variations in vasomotor function tends to increase tumor perfusion.	(Voss et al., 2019)
Liver cancer	Clinical trial	According to this recent research, the overexpression of eNOS and iNOS in cancer tissue, but not nNOS, is a factor that leads to elevated NO levels in patients with HCC.	(Zhang et al., 2019)

### 6.5 Hepatocellular carcinoma

**Figure 3:**
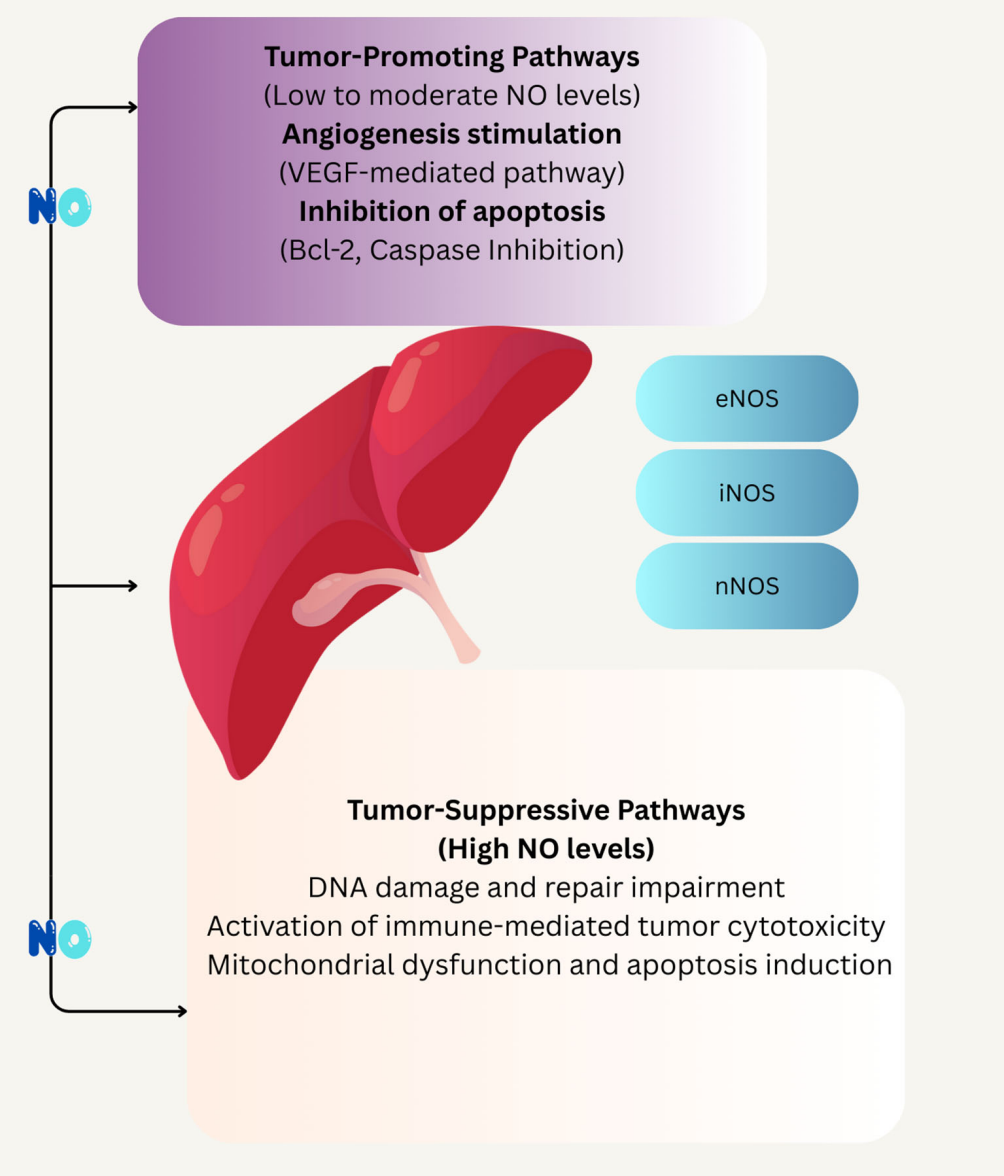
Schematic illustrates of the concentration-dependent effects of NO on tumor progression and suppression. At low to moderate NO levels, tumor-promoting pathways are activated, including VEGF-mediated angiogenesis and inhibition of apoptosis through Bcl-2 and caspase pathways. In contrast, high NO levels trigger tumor-suppressive mechanisms such as DNA damage, impaired repair, immune-mediated cytotoxicity, and mitochondrial dysfunction leading to apoptosis. The involvement of different NOS isoforms eNOS, iNOS, and nNOS s highlighted in mediating these divergent outcomes.

Hepatocellular carcinoma (HCC) is the second most common cause of cancer-related mortality worldwide. NOS expression and autophagy inhibition have been linked to cancer cell death (**Figure 3**) (Al-Shahari et al., 2022). Lei Zhou et al., in a 2012 study, indicated that decreased levels of NO/NOS-2 in liver tissues may be relevant to the development and metastasis of HCC. It was also found that the level of NO was higher in HCC tissues without metastasis than in those with metastasis (Zhou et al., 2012). In a 2016 study, Lei Zhou et al. found that NO inhibits the proliferation of HepG2 cells, with more significant suppression when using increasing concentrations of sodium nitroprusside (SNP), and NO stopped HepG2 cells in the G1 phase. The results indicated that cell apoptosis depends on NO concentration. NO suppresses proliferation, migration, and invasion, stops the cell cycle, and induces apoptosis in HepG2 cells; however, the detailed mechanism is still unclear, and further investigation is required (Zhou et al., 2016).

### 6.6 Gastric cancer

*H. pylori* is a Gram-negative bacterium colonizing the human gastric mucosa (Kim et al., 2011). *H. pylori* is challenged by a toxic environment that produces reactive oxygen species, such as hydrogen peroxide and superoxide anions, and reactive nitrogen species, most notably NO (Allen et al., 2023). This bacterium is mainly responsible for persistent oxidative stress in the stomach and induction of chronic immune responses, resulting in DNA damage and gastric cancer (also called stomach cancer). Oxidative stress results from the excessive release of ROS/RNS by activated neutrophils, whereas bacteria also produce ROS in host cells (Jain et al., 2021). Elevated NO is caused by up-regulated iNOS following inflammation or tissue damage in gastric epithelial cells. Increased iNOS expression in *H. pylori*-associated chronic gastritis diminishes and returns to baseline once it is eradicated (**Figure 3**) (Saaed et al., 2021). Gobert et al. reported that *H. pylori* expresses the gene rocF that encodes arginase, effectively dampening NO production by iNOS in macrophages (Gobert et al., 2001). Also, NOS2 expression is seen to increase in gastric adenocarcinoma. In a 2017 study, Oliveira et al. recognized 12 miRNAs associated with NOS2 gene expression in gastric carcinoma (**Table 2**) (de Oliveira et al., 2017).

### 6.7 Pancreatic cancer

Pancreatic ductal adenocarcinoma (PDAC) is one of the deadliest epithelial malignancies in the world (Connor and Gallinger, 2022). Excessive NO production and the expression of iNOS are closely related to PDAC, promoting the occurrence of cancer and angiogenesis (Xiong et al., 2021). A study conducted in 2003 on PDAC patients found that the expression rate of iNOS was 62.7%, which was related to lymph node metastasis (Liu et al., 2003). In another study in 2016, Jian Wang et al. suggested that PDAC expresses a high level of NOS2, leading to sustained NO production. Also, enhanced NOS2 expression in tumors was significantly associated with poor survival in PDAC patients (Wang et al., 2016a). Although less directly exposed to oral microbial products, pancreatic cancer may be influenced by systemic NO derived from dietary NO_3_^-^metabolism, which contributes to the body’s NO pool. This systemic NO could reach the pancreas via circulation or ductal connections with the duodenum, potentially affecting tumor angiogenesis and progression. While the connection is less pronounced than in gastrointestinal cancers, dysbiosis altering NO_2_^-^ production may still play a subtle role in pancreatic ductal adenocarcinoma (Wang et al., 2016b).

### 6.8 Bile duct cancers

Biliary tract cancers (BTC), including intrahepatic cholangiocarcinoma (iCCA), extrahepatic cholangiocarcinoma (eCCA), and gallbladder carcinoma (GBC), account for approximately 3% of gastrointestinal malignancies (Fava et al., 2007). NO and inducible iNOS play significant roles in BTC pathogenesis, particularly through their regulation of Notch signaling, a critical pathway in bile duct development and carcinogenesis.

In cholangiocytes, inflammatory cytokines (e.g., TNF-α, IL-1β) induce iNOS expression, leading to elevated NO production, which promotes malignant transformation (Fava et al., 2007). NO regulates Notch signaling through multiple mechanisms. First, NO mediates S-nitrosylation of Notch receptors or ligands, stabilizing the Notch intracellular domain (NICD) and enhancing its nuclear translocation to activate oncogenic target genes like *HES1* and *HEY1 (* Charles et al., 2010). Second, NO upregulates Notch-1 expression by activating transcription factors such as NF-κB, which is heightened in the inflammatory tumor microenvironment of cholangiocarcinoma (Ma et al., 2013). Third, NO-induced nitrosative stress impairs DNA repair mechanisms, leading to epigenetic changes (e.g., histone modifications) that further amplify Notch signaling, promoting tumor progression (Khan et al., 2020).

Notch-1 is hyper-expressed in cholangiocarcinoma cells, correlating with tumor growth and invasion, and its dependency on NO generation positions NO as a key regulator of BTC pathogenesis (Fava et al., 2007). The oral microbiome may indirectly contribute to this process, as NO derived from dietary NO_3_^-^metabolism by bacteria like *Veillonella* and *Rothia* increases systemic NO levels, potentially enhancing Notch activation in tumors (Hyde et al., 2014). Targeting NO-Notch interactions, such as with iNOS or Notch inhibitors, holds therapeutic promise for BTC, warranting further clinical exploration.

### 6.9 Esophageal cancer

Esophageal cancer is one of the most fatal malignancies in the world. Esophageal SCC and esophageal adenocarcinoma (EAC) are the two main types of esophageal cancer (Shi and Chen, 2022). Esophageal adenocarcinoma involves chronic exposure of the distal epithelium to stomach and bile acids, causing inflammation and intestinal metaplasia, which is also known as Barrett’s esophagus (BE). The esophageal injury can be caused by reflux directly or by the generation of ROS indirectly. Other risk factors for EAC include obesity and gastroesophageal reflux disease (GERD). The gram-negative, dysbiotic microbiota in the EAC may stimulate iNOS, leading to lower esophageal sphincter relaxation and inducing GERD. NO, and iNOS induce apoptosis, angiogenesis, and DNA damage during tumorigenesis in esophageal adenocarcinoma. Studies have also shown high iNOS expression in BE patients (Sharma et al., 2022).

NOS2 expression is upregulated in esophageal cancer. Mutations in the NOS2 gene or its promoter are related to NOS induction and increased rates of different cancers, including esophageal cancer. Also, mutations of the p53 gene are seen in this malignancy (de Oliveira et al., 2017).

Esophagus (SCC) is characterized by increased expression of genes encoding glucose transporter 1 (GLUT1), iNOS, and ornithine decarboxylase (ODC) of the L-arginine/NO/polyamine pathway (Bednarz-Misa et al., 2020). Increased esophageal (SCC) rates are also associated with specific changes in the salivary microbiota (Chen et al., 2015). An increase in harmful bacteria shifts the balance towards a disease state. An imbalanced microbiota environment, also known as dysbiosis, leads to immune activation and chronic inflammation of the esophageal mucosa (**Table 2**) (Chiang et al., 2023).

### 6.10 Glioblastoma

Gliomas represent the most prevalent form of brain tumors, among which glioblastomas are the most malignant subtype. Despite advances in comprehending their biology and treatment strategies, median survival remains disappointingly low. Inflammatory processes involving NO critically contribute to glioma formation. The inducible isoform iNOS is highly overexpressed in gliomas and has been linked to resistance against temozolomide (TMZ) treatment, neoplastic transformation, and modulation of immune response. At low levels, NO enhances tumor growth, while high levels may induce cytotoxicity, reflecting its dual nature

**Figure 4:**
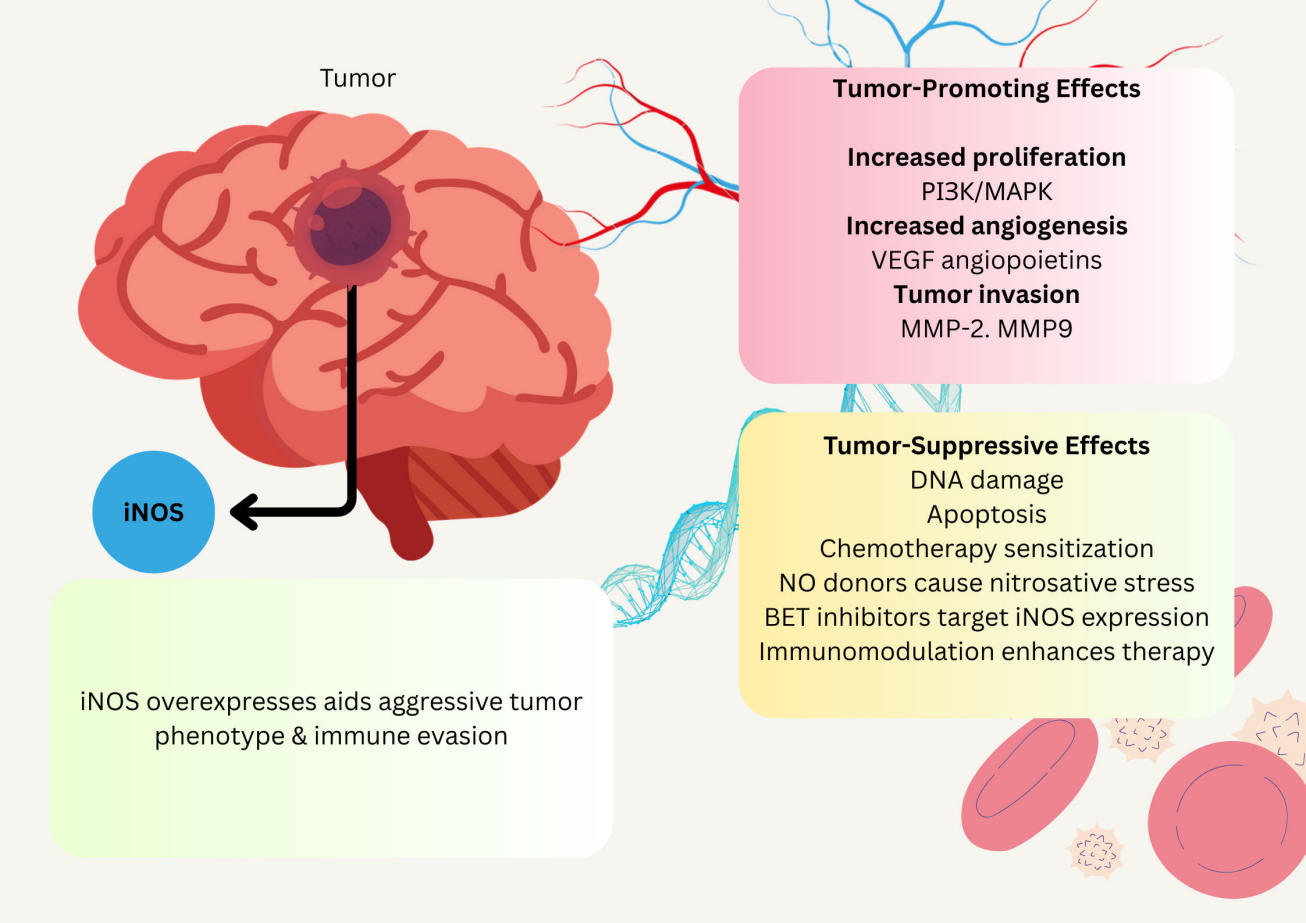
Schematic illustration of NO association effect on glioblastoma. NO administration affects glioblastoma by slowing their growth and enhancing their susceptibility to chemotherapy. This has potential implications for long-term management. Additionally, BET inhibitors targeting iNOS expression in cancer cells can significantly improve therapeutic efficacy in glioblastoma.

While both *in vitro* and *in vivo* studies showed the potential of iNOS inhibitors as effective treatments for gliomas, no clinical trials on gliomas have been published (**Figure 4**) (Mazurek and Rola, 2021; Tripathi et al., 2023).

Merenzone et al.’s systematic review demonstrated that inhibitors exhibit substantial potential as treatment options for oncologic lesions, and they have demonstrated a safe toxicity profile in humans for other pathological conditions. Their research endeavors should be focused on investigating their potential effects on brain tumors (Merenzon et al., 2023). While glioblastoma’s connection to the oral microbiome is less direct, systemic NO levels partly derived from dietary NO_3_^-^metabolism may still be relevant. As circulating NO could influence the tumor microenvironment or immune responses in the brain. Although endogenous NO production via iNOS dominates in glioblastoma, microbiome-derived NO might subtly modulate systemic inflammation, warranting further exploration of this indirect link (Fahey et al., 2018).

### 6.11 Triple-negative breast cancer

**Figure 5:**
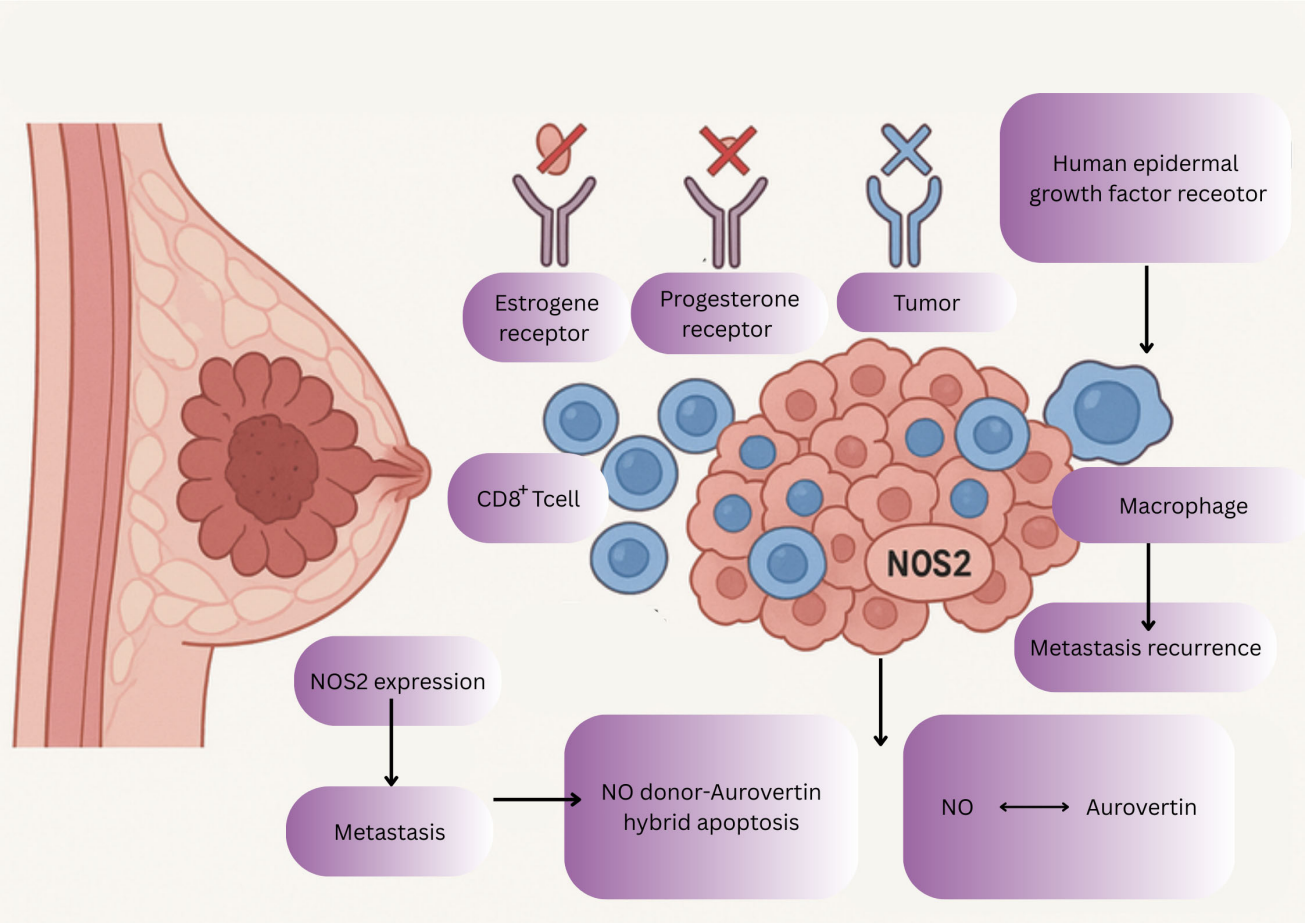
Illustration highlights the aggressive nature of TNBC, characterized by the absence of ER, and PR. High NOS2 expression promotes tumor progression, metastasis, and poor prognosis. The diagram depicts immune modulation through NOS2 depletion and COX2 inhibition, enhancing CD8^+^ T-cell infiltration and anti-tumor response. It also shows the therapeutic potential of NO donor–aurovertin B hybrids in inducing apoptosis and suppressing tumor growth.

This type of breast is an aggressive subtype occurring more in younger woman, characterized by rapid metastasis, a poor prognosis, absence of estrogen receptors (ER), progesterone receptors (PR), and human epidermal growth factor receptor expression (Obidiro et al., 2023). NOS2 plays a significant role in TNBC progression as high levels of NOS2 expression correlates with poor prognosis, increased metastasis, and cancer recurrence (**Figure 5**) (Nafea et al., 2021).

One study suggests that reducing NOS2 expression alters the spatial orientation and density of lymphoid cells, particularly CD8 T cells within the tumor microenvironment and when NOS2 is depleted and COX2 is inhibited, tumor growth slows and the immune response enhances (Somasundaram et al., 2022).

Another study focuses on NO donors and shows that NO donor-aurovertin hybrids can be used to treat TNBC by inducing apoptosis. These hybrids combine aurovertin B (AVB), a natural polyketide with anti-proliferative effects, with NO donors to enhance therapeutic efficacy (**Figure 5**) (Ma et al., 2024).

### 6.12 Nitric oxide and NOS2 as prognostic markers in cancer

High levels of NO and iNOS have been identified as markers of poor prognosis in several cancers, reflecting their role in promoting tumor progression, angiogenesis, and treatment resistance.

In PDAC, elevated NOS2 expression is strongly associated with poor survival. A study of PDAC patients found that 62.7% of tumors expressed high NOS2 levels, correlating with lymph node metastasis and reduced overall survival (Wang et al., 2016b). This suggests that NOS2-driven NO production enhances tumor aggressiveness, making it a robust prognostic marker. For CRC, NOS2 expression is observed in 50–60% of cases, with high levels linked to worse prognosis, particularly in advanced stages. Patients with elevated NOS2 expression exhibit increased tumor invasiveness and reduced survival, likely due to NO-mediated inflammation and angiogenesis (Monteiro et al., 2019). This positions NOS2 as a marker of poor outcomes in CRC. Also, HCC, overexpression of iNOS and endothelial eNOS contributes to elevated NO levels, associated with tumor progression and poorer prognosis. Clinical studies show that high iNOS expression correlates with increased tumor size, vascular invasion, and reduced survival in HCC patients (Zhang et al., 2019). NO’s role in promoting angiogenesis and inhibiting apoptosis underscores its prognostic relevance.

Glioblastoma also demonstrates high iNOS expression as a marker of poor prognosis. Elevated iNOS levels drive resistance to temozolomide and enhance tumor growth, correlating with shorter survival times in preclinical and clinical (Merenzon et al., 2023).

In gastric cancer, the prognostic role of NOS2 is less consistent but notable in advanced cases. High iNOS expression is associated with larger tumors (>5 cm), serosal invasion, and lymph node metastases, linked to reduced survival (Rajnakova et al., 2001). However, some studies report variability, suggesting context-dependent effects. In other cancers, such as esophageal cancer and bile duct cancers, evidence is less conclusive. While iNOS expression is elevated in esophageal (SCC) and cholangiocarcinoma, its direct correlation with poor prognosis is not consistently reported, warranting further investigation (Suksawat et al., 2017; Bednarz-Misa et al., 2020).

In summary, high NO/NOS2 levels serve as markers of poor prognosis in pancreatic, colorectal, hepatocellular, glioblastoma, and, to a lesser extent, gastric cancers. These associations reflect NO’s role in enhancing tumor aggressiveness and resistance to therapy. Future studies should validate these markers in larger cohorts and explore their integration into clinical prognostic models, potentially guiding NO-targeted therapies.

## 7 Nitric oxide-based anti-cancer therapies

Four major categories of NO-based anti-cancer therapies—NO donors, phosphodiesterase inhibitors (PDE-i), soluble guanylyl cyclase (sGC) activators, and immunomodulators NO to target cancer cells, offering promising avenues for treatment (Mintz et al., 2021). NO donors, such as glyceryl trinitrate, release NO directly into the tumor microenvironment, inducing cytotoxicity by disrupting mitochondrial function and sensitizing cancer cells to chemotherapy and radiotherapy (Huang et al., 2017). Phosphodiesterase inhibitors, like sulindac sulfone, block cGMP degradation, enhancing NO-cGMP signaling to inhibit tumor cell proliferation and promote apoptosis (Mehta and Patel, 2019). Soluble guanylyl cyclase activators, such as riociguat, stimulate cGMP production, amplifying NO signaling to suppress tumor growth by inhibiting proliferation and inducing apoptosis, particularly in resistant cancers (Schenk et al., 2021). Immunomodulators, including TLR7 agonists combined with iNOS inhibitors, modulate NO levels to enhance cytotoxic T-cell and macrophage activity, boosting immune-mediated tumor cell killing (PeÑarando et al., 2019). These mechanisms, detailed in the following subsections, highlight NO’s therapeutic potential, potentially modulated by oral microbiome-derived NO from dietary NO_3_^-^ (Morou-Bermúdez et al., 2022).

### 7.1 NO donors

NO donors are compounds capable of generating NO, including Glyceryl triNO_3_^-^(GTN) (Huang et al., 2017). They function by elevating NO or NO isoforms without endogenous production (Mintz et al., 2021). NO donors work through different mechanisms, i.e., increased NO concentration within tissue beds (Alimoradi et al., 2019). In cancer therapy, NO donors can work alone, combined with other therapeutics, such as chemo–, radio–, or immunotherapy, and hybridization of NO donors. Recently, organic NO_3_^-^ has joined the clinical field of cancer treatment and affects tumor vessels by improving tumor oxygenation (Huang et al., 2017). In a 2015 phase I study, Illum et al. used the combination of 5-FU, topical GTN, and radiation therapy against advanced operable rectal cancer to evaluate the maximum tolerable dose of NO donor (Illum et al., 2015). Planchette et al.’s study shows that NO can potentially bypass chemotherapy resistance mechanisms associated with platinum-based drugs. Research has focused on combining platinum chemotherapeutic drugs with NO donors. These combination regimens have demonstrated antitumor benefits, particularly in non-small cell lung cancer (Plenchette et al., 2017). Phosphodiesterase is a group of iso-enzymes that hydrolyze cyclic nucleotides and lowering intracellular cAMP and cGMP, leading to tumorigenic effects. Many tumors show a decreased level of intracellular cAMP due to high PDE expression. For example, Sulindac sulfone (exisulind) inhibits PDE2 and PDE5 isoforms and induces apoptosis in colon cancer cells (Mehta and Patel, 2019). Soluble guanylate cyclase (sGC) is a protein essential for sensing NO and is upregulated in disease progression, leading to chemoresistance. Genetic reduction of sGC levels or pharmacological inhibition of sGC activity with an NOS inhibitor re-sensitized a progression in treatment. In a 2021 study, Schenk et al. concluded that the sGC signaling pathway can be the reason for relapsing small cell lung cancer (SCLC) (Schenk et al., 2021).

### 7.2 Immunomodulators

Immunomodulators represent a promising class of NO-based anti-cancer therapies, leveraging NO’s role in modulating the immunosuppressive tumor microenvironment to enhance immune responses. This subsection explores how NO-targeted immunomodulators can improve cancer immunotherapy, with potential links to microbiome-mediated NO production.

Activated macrophages generate NO or have cytotoxic antitumor activity, indicating NO’s pivotal role in the immune system. NO plays a vital role in the immunosuppressive tumor environment; therefore, therapies against NO have been considered in different types of tumors to increase the efficacy of immunotherapy (PeÑarando et al., 2019). For example, administering the Toll-like receptor 7 (TLR7) agonist imiquimod requires the simultaneous inhibition of iNOS activity to promote tumor antigen-specific Th1 responses and suppress tumor growth *in vivo* (Ito et al., 2015). Arginine is an amino acid used in protein, cell division, and wound healing biosynthesis that can be obtained from daily food intake or synthesized in the body. Arginine-dependent migration requires arginine to be metabolized by the NO synthase enzyme (NOS) (Al-Koussa et al., 2020). Feng et al.’s study on glioblastoma, based on mRNA sequencing data of 560 IDH-wildtype glioblastoma patients from three public cohorts, aimed to construct an arginine metabolism-related genes signature (ArMRS) based on four essential arginine metabolism-related genes (ArMGs). Analyses of tumor immune microenvironment revealed that higher ArMRS was correlated with more immune infiltration and a relatively “hot” immunological phenotype. Also, demonstrated that ArMRS was positively correlated with the expression of multiple immunotherapy targets, including PD1 and B7-H3. Additionally, the glioblastomas in the ArMRS high-risk group would present with more cytotoxic T cells (CTLs) infiltration and better-predicted response to immune checkpoint inhibitors (ICIs) (Feng et al., 2023).

The oral microbiome may indirectly influence these therapies by contributing to systemic NO levels through dietary NO_3_^-^metabolism. Nitrate-reducing bacteria (*Veillonella*, *Rothia*) produce NO_2_^-^, which can increase circulating NO, potentially modulating immune responses in tumors (Hyde et al., 2014). Future research should explore how microbiome-targeted interventions could optimize NO levels for immunotherapy.

In summary, NO-based immunomodulators offer a novel approach to overcome tumor immunosuppression, with potential synergies with microbiome-derived NO. Clinical trials are needed to validate these strategies and integrate them with existing immunotherapies.

## 8 Future prospects

As research into the human microbiome and its influence on NO production advances, the complex relationship between dietary NO_3_^-^, the oral microbiome, and cancer risk emerges as a pivotal area for future investigation. Dietary NO_3_^-^, primarily derived from vegetables and processed meats, exhibits a dual role in cancer biology, as discussed, they can either protect against or promote carcinogenesis, depending on their source and the physiological context. This duality underscores the need for a nuanced understanding to inform cancer prevention and therapeutic strategies. Evidence suggests that NO_3_^-^ from vegetables, which are accompanied by antioxidants such as vitamin C, generally exert protective effects against gastrointestinal cancers, including gastric and CRC, by inhibiting the formation of carcinogenic NOCs (Song et al., 2015). Conversely, NO_2_^-^ from processed meats, which lack these protective compounds and are rich in amines, facilitates NOC formation, elevating cancer risk (Said Abasse et al., 2022). However, the tipping point at which dietary NO_3_^-^ transitions from beneficial to harmful remains poorly defined (Li et al., 2013). Conditions like acidic gastric pH, *H. pylori* infection, or atrophic gastritis can enhance NOC synthesis from NO_2_^-^s (Bryan et al., 2012). Genetic variations affecting inflammation or DNA repair may further amplify susceptibility to NOCs (Mubayi et al., 2011).

While a broad consensus supports the protective role of vegetable-derived NO_3_^-^ and the risks posed by processed meat NO_2_^-^s, inconsistencies persist. Some studies find no clear link between NO_3_^-^intake and cancer risk, particularly for NO_3_^-^ from drinking water, suggesting a need for improved dietary assessment methods and control of confounding variables (Xie et al., 2016). Additionally, the safe thresholds for NO_3_^-^ and NO_2_^-^ consumption remain contentious, necessitating further research to establish evidence-based limits. To address these uncertainties, future studies should prioritize the following: mixed genomics, transcriptomics, proteomics, and metabolomics to get the full picture of NO in cancer. That could uncover new targets and show us how NO teams up with things like reactive oxygen species or cytokines maybe even spark some clever combo treatments. Further steps could include modeling blend NO data with a patient’s tumor stage, microbial mix, and genetics. Which could fine-tune NO donor doses or pair them with immune checkpoint inhibitors think glioblastoma, where arginine and immunotherapy overlap. That’s personalized medicine in action.

These steps feel like the natural next move. They build on what we’ve learned and push us closer to making NO a real tool against cancer. The way it ties into the microbiome and therapy, it’s a wide-open field.

## 9 Conclusion

The intricate relationship between NO, the human microbiome, and cancer pathogenesis, as elucidated in this review, underscores NO’s remarkable dual nature as both a promoter and suppressor of tumor development. It’s a molecule that wears two hats one minute it’s nudging tumors to grow, the next it’s putting the brakes on them. We’ve dug into how NO gets made, whether through the L-arginine-NO synthase NOS pathway or the NO_3_^-^NO_2_^-^NO pathway route powered by the oral microbiome. That link between what we eat, the bacteria in our mouths, and cancer risk? It’s honestly pretty fascinating. What stands out is how NO plays out differently depending on the situation. In cancers like gastric, esophageal, colorectal, pancreatic, and even glioblastoma, it’s a bit of a wildcard. At lower doses, it’s like a cheerleader for tumor growth, helping blood vessels form and making cancer cells bigger. But crank up the levels, and NO flips the script, triggering cell death and hinting at a built-in defense against tumors. The oral microbiome is a big piece of this puzzle, turning dietary NO_3_^-^ into NO and tying our daily habits straight to cancer outcomes. Ideas like NO donors, phosphodiesterase inhibitors, soluble guanylyl cyclase activators, and immunomodulators could shake up current treatments, maybe even help patients beat drug resistance or live longer, as some clinical studies backing this up, showing how NOS levels and NO activity shift in cancer tissues and track with how the disease progresses. Then there’s the tumor microenvironment, where NO interacts with immune cells, stromal bits, and oxidative stress in ways that are, frankly, a little mind-boggling. It’s this complexity that makes NO so intriguing, it could either fuel cancer or give us a way to stop it, depending on how we approach it. The clinical patterns talked about, like how NOS ties to prognosis, make me think we’re onto something big, like personalized treatments tailored to each patient’s unique setup.

This review shines a light on NO’s double-edged role in cancer and gets us thinking about how to turn that into real-world solutions. It’s a field brimming with potential, and I’d say we’ve laid a solid foundation for what’s next.

## References

[B1] Alaei L. Izadi Z. Jafari S. Jahanshahi F. Jaymand M. Mohammadi P.. (2021). Irreversible thermal inactivation and conformational lock of alpha glucosidase. J. Biomolecular Structure Dynamics. 39, 3256–3262. doi: 10.1080/07391102.2020.1762742 32345145

[B2] Alimoradi H. Greish K. Gamble A. B. Giles G. I. (2019). Controlled delivery of nitric oxide for cancer therapy. Pharm. Nanotechnology. 7, 279–303. doi: 10.2174/2211738507666190429111306 PMC6967185 31595847

[B3] Al-Koussa H. El Mais N. Maalouf H. Abi-Habib R. El-Sibai M. (2020). Arginine deprivation: A potential therapeutic for cancer cell metastasis? A review. Cancer Cell Int. 20, 1–7. doi: 10.1186/s12935-020-01232-9 32390765 PMC7201942

[B4] Allen M. G. Bate M. Y. Tramonte L. M. Avalos E. Y. Loh J. Cover T. L.. (2023). Regulation of helicobacter pylori urease and acetone carboxylase genes by nitric oxide and the crdRS two-component system. Microbiol. Spectr. 11 (1), e04633–e04622. doi: 10.1128/spectrum.04633-22 36625670 PMC9927306

[B5] Alnuaimi A. D. Wiesenfeld D. O’Brien-Simpson N. M. Reynolds E. C. McCullough M. J. (2015). Oral Candida colonization in oral cancer patients and its relationship with traditional risk factors of oral cancer: a matched case-control study. Oral. Oncol. 51, 139–145. doi: 10.1016/j.oraloncology.2014.11.008 25498921

[B6] Al-Shahari E. A. El Barky A. R. Mohamed T. M. Abdelaal K. Alm-Eldeen A. A. (2022). L-arginine is potential than doxorubicin or their combination in improving hepatocellular carcinoma-induced liver nitric oxide overproduction and arginase expression downregulation. Fresenius Environ. Bulletin. 31, 4572–4580.

[B7] Araujo-Gutierrez R. Van Eps J. Kirui D. Bryan N. Kang Y. Fleming J.. (2019). Enhancement of gemcitabine cytotoxicity in pancreatic adenocarcinoma through controlled release of nitric oxide. Biomed. Microdevices. 21, 1–8. doi: 10.1007/s10544-019-0375-z 30790060

[B8] Arweiler N. B. Netuschil L. (2016). “The oral microbiota,” in Microbiota of the human body: implications in health and disease, 45–60. (Switzerland: Springer, Cham).

[B9] Bedale W. Sindelar J. J. Milkowski A. L. (2016). Dietary nitrate and nitrite: Benefits, risks, and evolving perceptions. Meat Sci. 120, 85–92. doi: 10.1016/j.meatsci.2016.03.009 26994928

[B10] Bednarz-Misa I. Fortuna P. Fleszar M. G. Lewandowski Ł. Diakowska D. Rosińczuk J.. (2020). Esophageal squamous cell carcinoma is accompanied by local and systemic changes in L-arginine/NO pathway. Int. J. Mol. Sci. 21, 6282. doi: 10.3390/ijms21176282 32872669 PMC7503331

[B11] Belkaid Y. Hand T. W. (2014). Role of the microbiota in immunity and inflammation. Cell. 157, 121–141. doi: 10.1016/j.cell.2014.03.011 24679531 PMC4056765

[B12] Bhalodi A. A. van Engelen T. S. R. Virk H. S. Wiersinga W. J. (2019). Impact of antimicrobial therapy on the gut microbiome. J. Antimicrobial Chemotherapy 74, i6–i15. doi: 10.1093/jac/dky530 PMC6382031 30690540

[B13] Blekkenhorst L. C. Bondonno N. P. Liu A. H. Ward N. C. Prince R. L. Lewis J. R.. (2018). Nitrate, the oral microbiome, and cardiovascular health: a systematic literature review of human and animal studies. Am. J. Clin. Nutr. 107, 504–522. doi: 10.1093/ajcn/nqx046 29635489

[B14] Bray F. Ferlay J. Soerjomataram I. Siegel R. L. Torre L. A. Jemal A. (2018). Global cancer statistics 2018: GLOBOCAN estimates of incidence and mortality worldwide for 36 cancers in 185 countries. CA Cancer J. Clin. 68, 394–424. doi: 10.3322/caac.21492 30207593

[B15] Bryan N. S. Alexander D. D. Coughlin J. R. Milkowski A. L. Boffetta P. (2012). Ingested nitrate and nitrite and stomach cancer risk: An updated review. Food Chem. Toxicology. 50, 3646–3665. doi: 10.1016/j.fct.2012.07.062 22889895

[B16] Bryan N. S. Burleigh M. C. Easton C. (2022). The oral microbiome, nitric oxide and exercise performance. Nitric. Oxide 125, 23–30. doi: 10.1016/j.niox.2022.05.004 35636654

[B17] Cali F. Cantone M. Cosentino F. I. I. Lanza G. Ruggeri G. Chiavetta V.. (2019). Interpreting genetic variants: hints from a family cluster of parkinson’s disease. J. Parkinsons Dis. 9, 203–206. doi: 10.3233/JPD-171292 30400105

[B18] Camañes-Gonzalvo S. Montiel-Company J. M. Lobo-de-Mena M. Safont-Aguilera M. J. Fernández-Diaz A. López-Roldán A.. (2024). Relationship between oral microbiota and colorectal cancer: A systematic review. J. Periodontal Res. 59, 1071–1082. doi: 10.1111/jre.13289 38775019 PMC11626693

[B19] Caselli E. Fabbri C. D’Accolti M. Soffritti I. Bassi C. Mazzacane S.. (2020). Defining the oral microbiome by whole-genome sequencing and resistome analysis: the complexity of the healthy picture. BMC Microbiol. 20, 1–19. doi: 10.1186/s12866-020-01801-y 32423437 PMC7236360

[B20] Charles N. Ozawa T. Squatrito M. Bleau A. M. Brennan C. W. Hambardzumyan D.. (2010). Perivascular nitric oxide activates notch signaling and promotes stem-like character in PDGF-induced glioma cells. Cell Stem Cell. 6, 141–152. doi: 10.1016/j.stem.2010.01.001 20144787 PMC3818090

[B21] Chen X. Winckler B. Lu M. Cheng H. Yuan Z. Yang Y.. (2015). Oral microbiota and risk for esophageal squamous cell carcinoma in a high-risk area of China. PloS One 10, e0143603. doi: 10.1371/journal.pone.0143603 26641451 PMC4671675

[B22] Chiang H. C. Hughes M. Chang W. L. (2023). The role of microbiota in esophageal squamous cell carcinoma: A review of the literature. Thorac. Cancer. 14, 2821–2829. doi: 10.1111/1759-7714.15096 37675608 PMC10542467

[B23] Colla G. Kim H.-J. Kyriacou M. C. Rouphael Y. (2018). Nitrate in fruits and vegetab les. Scientia Horticulturae. 237, 221–238. doi: 10.1016/j.scienta.2018.04.016

[B24] Connor A. A. Gallinger S. (2022). Pancreatic cancer evolution and heterogeneity: integrating omics and clinical data. Nat. Rev. Cancer. 22, 131–142. doi: 10.1038/s41568-021-00418-1 34789870

[B25] Dashper S. Mitchell H. Lê Cao K.-A. Carpenter L. Gussy M. Calache H.. (2019). Temporal development of the oral microbiome and prediction of early childhood caries. Sci. Rep. 9, 19732. doi: 10.1038/s41598-019-56233-0 31874981 PMC6930300

[B26] de Oliveira G. A. Cheng R. Y. Ridnour L. A. Basudhar D. Somasundaram V. McVicar D. W.. (2017). Inducible nitric oxide synthase in the carcinogenesis of gastrointestinal cancers. Antioxidants Redox Signaling 26, 1059–1077. doi: 10.1089/ars.2016.6850 27494631 PMC5488308

[B27] Derakhshankhah H. Izadi Z. Alaei L. Lotfabadi A. Saboury A. A. Dinarvand R.. (2017). Colon cancer and specific ways to deliver drugs to the large intestine. Anti-Cancer Agents Medicinal Chem. (Formerly Curr. Medicinal Chemistry-Anti-Cancer Agents). 17, 1317–1327. doi: 10.2174/1871520617666170213142030 28270073

[B28] Douaiher J. Ravipati A. Grams B. Chowdhury S. Alatise O. Are C. (2017). Colorectal cancer-global burden, trends, and geographical variations. J. Surg. Oncol. 115, 619–630. doi: 10.1002/jso.v115.5 28194798

[B29] Fahey J. M. Stancill J. S. Smith B. C. Girotti A. W. (2018). Nitric oxide antagonism to glioblastoma photodynamic therapy and mitigation thereof by BET bromodomain inhibitor JQ1. J. Biol. Chem. 293, 5345–5359. doi: 10.1074/jbc.RA117.000443 29440272 PMC5892570

[B30] Fan Y. Pedersen O. (2021). Gut microbiota in human metabolic health and disease. Nat. Rev. Microbiol. 19, 55–71. doi: 10.1038/s41579-020-0433-9 32887946

[B31] Fava G. Marzioni M. Benedetti A. Glaser S. DeMorrow S. Francis H.. (2007). Molecular pathology of biliary tract cancers. Cancer Letters. 250, 155–167. doi: 10.1016/j.canlet.2006.09.011 17069969

[B32] Feng W. Zuo M. Li W. Chen S. Wang Z. Yuan Y.. (2023). A novel score system based on arginine metabolism-related genes to predict prognosis, characterize immune microenvironment, and forecast response to immunotherapy in IDH-wildtype glioblastoma. Front. Pharmacol. 14. doi: 10.3389/fphar.2023.1145828 PMC10196947 37214463

[B33] Ferlay J. Soerjomataram I. Dikshit R. Eser S. Mathers C. Rebelo M.. (2015). Cancer incidence and mortality worldwide: sources, methods and major patterns in GLOBOCAN 2012. Int. J. cancer. 136, E359–EE86. doi: 10.1002/ijc.v136.5 25220842

[B34] Ferysiuk K. Wójciak K. M. (2020). Reduction of nitrite in meat products through the application of various plant-based ingredients. Antioxidants. 9, 711. doi: 10.3390/antiox9080711 32764511 PMC7464959

[B35] Franzosa E. A. Morgan X. C. Segata N. Waldron L. Reyes J. Earl A. M.. (2014). Relating the metatranscriptome and metagenome of the human gut. Proc. Natl. Acad. Sci. U S A. 111, E2329–E2338. doi: 10.1073/pnas.1319284111 24843156 PMC4050606

[B36] Gaetti-Jardim E. Marcelino S. L. Feitosa A. C. R. Romito G. A. Avila-Campos M. J. (2009). Quantitative detection of periodontopathic bacteria in atherosclerotic plaques from coronary arteries. J. Med. Microbiol. 58, 1568–1575. doi: 10.1099/jmm.0.013383-0 19679682

[B37] Gago B. Nyström T. Cavaleiro C. Rocha B. S. Barbosa R. M. Laranjinha J.. (2008). The potent vasodilator ethyl nitrite is formed upon reaction of nitrite and ethanol under gastric conditions. Free Radical Biol. Med. 45, 404–412. doi: 10.1016/j.freeradbiomed.2008.04.027 18482590

[B38] Gobert A. P. McGee D. J. Akhtar M. Mendz G. L. Newton J. C. Cheng Y.. (2001). *Helicobacter pylori* arginase inhibits nitric oxide production by eukaryotic cells: A strategy for bacterial survival. Proc. Natl. Acad. Sci. 98, 13844–13849. doi: 10.1073/pnas.241443798 11717441 PMC61129

[B39] Gonzalez M. Clayton S. Wauson E. Christian D. Tran Q. K. (2025). Promotion of nitric oxide production: mechanisms, strategies, and possibilities. Front. Physiol. 16, 1545044. doi: 10.3389/fphys.2025.1545044 39917079 PMC11799299

[B40] He J. Li Y. Cao Y. Xue J. Zhou X. (2015). The oral microbiome diversity and its relation to human diseases. Folia Microbiol. (Praha). 60, 69–80. doi: 10.1007/s12223-014-0342-2 25147055

[B41] Helmink B. A. Khan M. W. Hermann A. Gopalakrishnan V. Wargo J. A. (2019). The microbiome, cancer, and cancer therapy. Nat. Med. 25, 377–388. doi: 10.1038/s41591-019-0377-7 30842679

[B42] Hezel M. Weitzberg E. (2015). The oral microbiome and nitric oxide homoeostasis. Oral. diseases. 21, 7–16. doi: 10.1111/odi.2014.21.issue-1 23837897

[B43] Hofseth L. J. Hussain S. P. Wogan G. N. Harris C. C. (2003). Nitric oxide in cancer and chemoprevention. Free Radical Biol. Med. 34, 955–968. doi: 10.1016/S0891-5849(02)01363-1 12684081

[B44] Hord N. G. Tang Y. Bryan N. S. (2009). Food sources of nitrates and nitrites: the physiologic context for potential health benefits. Am. J. Clin. Nutr. 90, 1–10. doi: 10.3945/ajcn.2008.27131 19439460

[B45] Hu Y. Xiang J. Su L. Tang X. (2020). The regulation of nitric oxide in tumor progression and therapy. J. Int. Med. Res. 48, 0300060520905985. doi: 10.1177/0300060520905985 32090657 PMC7110915

[B46] Huang Z. Fu J. Zhang Y. (2017). Nitric oxide donor-based cancer therapy: advances and prospects. J. Med. Chem. 60, 7617–7635. doi: 10.1021/acs.jmedchem.6b01672 28505442

[B47] Huang J. Lucero-Prisno D. E. III Zhang L. Xu W. Wong S. H. Ng S. C.. (2023). Updated epidemiology of gastrointestinal cancers in East Asia. Nat. Rev. Gastroenterol. Hepatol. 20, 271–287. doi: 10.1038/s41575-022-00726-3 36631716

[B48] Huttenhower C. Gevers D. Knight R. Abubucker S. Badger J. H. Chinwalla A. T.. (2012). Structure, function and diversity of the healthy human microbiome. Nature 486, 207–214. 22699609 10.1038/nature11234 PMC3564958

[B49] Hyde E. R. Andrade F. Vaksman Z. Parthasarathy K. Jiang H. Parthasarathy D. K.. (2014). Metagenomic analysis of nitrate-reducing bacteria in the oral cavity: implications for nitric oxide homeostasis. PloS One 9, e88645. doi: 10.1371/journal.pone.0088645 24670812 PMC3966736

[B50] Ignarro L. J. (1990). Biosynthesis and metabolism of endothelium-derived nitric oxide. Annu. Rev. Pharmacol. Toxicol. 30, 535–560. doi: 10.1146/annurev.pa.30.040190.002535 2188578

[B51] Ijyuuin T. Umehara F. (2012). Case of Streptococcus salivarius bacteremia/meningoencephalitis leading to discovery of early gastric cancer. Rinsho Shinkeigaku= Clin. Neurology. 52, 360–363. doi: 10.5692/clinicalneurol.52.360 22688117

[B52] Illum H. Wang D. H. Dowell J. E. Hittson W. J. Torrisi J. R. Meyer J.. (2015). Phase I dose escalation trial of nitroglycerin in addition to 5-fluorouracil and radiation therapy for neoadjuvant treatment of operable rectal cancer. Surgery. 158, 460–465. doi: 10.1016/j.surg.2015.04.007 25964028

[B53] Ingham A. C. Kielsen K. Mordhorst H. Ifversen M. Aarestrup F. M. Müller K. G.. (2021). Microbiota long-term dynamics and prediction of acute graft-versus-host disease in pediatric allogeneic stem cell transplantation. Microbiome. 9, 1–28. doi: 10.1186/s40168-021-01100-2 34183060 PMC8240369

[B54] Ito H. Ando T. Ogiso H. Arioka Y. Seishima M. (2015). Inhibition of induced nitric oxide synthase enhances the anti-tumor effects on cancer immunotherapy using TLR7 agonist in mice. Cancer Immunology Immunother. 64, 429–436. doi: 10.1007/s00262-014-1644-6 PMC11029476 25567751

[B55] Izadi Z. Divsalar A. Saboury A. A. Sawyer L. (2016). β-lactoglobulin–pectin nanoparticle-based oral drug delivery system for potential treatment of colon cancer. Chem. Biol. Drug design. 88, 209–216. doi: 10.1111/cbdd.2016.88.issue-2 26896377

[B56] Jain U. Saxena K. Chauhan N. (2021). Helicobacter pylori induced reactive oxygen Species: A new and developing platform for detection. Helicobacter. 26, e12796. doi: 10.1111/hel.12796 33666321

[B57] Kamm A. Przychodzen P. Kuban-Jankowska A. Jacewicz D. Dabrowska A. M. Nussberger S.. (2019). Nitric oxide and its derivatives in the cancer battlefield. Nitric. Oxide 93, 102–114. doi: 10.1016/j.niox.2019.09.005 31541733

[B58] Kandalai S. Li H. Zhang N. Peng H. Zheng Q. (2023). The human microbiome and cancer: a diagnostic and therapeutic perspective. Cancer Biol. Ther. 24, 2240084. doi: 10.1080/15384047.2023.2240084 37498047 PMC10376920

[B59] Karpiński T. M. (2019). Role of oral microbiota in cancer development. Microorganisms. 7, 20. doi: 10.3390/microorganisms7010020 30642137 PMC6352272

[B60] Karwowska M. Kononiuk A. (2020). Nitrates/nitrites in food—Risk for nitrosative stress and benefits. Antioxidants. 9, 241. doi: 10.3390/antiox9030241 32188080 PMC7139399

[B61] Keller R. M. Beaver L. Prater M. C. Hord N. G. (2020). Dietary nitrate and nitrite concentrations in food patterns and dietary supplements. Nutr. Today 55, 218–226. doi: 10.1097/NT.0000000000000253

[B62] Kelly R. J. (2019). Emerging multimodality approaches to treat localized esophageal cancer. J. Natl. Compr. Canc Netw. 17, 1009–1014. doi: 10.6004/jnccn.2019.7337 31390584

[B63] Khan F. H. Dervan E. Bhattacharyya D. D. McAuliffe J. D. Miranda K. M. Glynn S. A. (2020). The role of nitric oxide in cancer: master regulator or not? Int. J. Mol. Sci. 21, 9393. doi: 10.3390/ijms21249393 33321789 PMC7763974

[B64] Kilian M. Chapple I. L. Hannig M. Marsh P. D. Meuric V. Pedersen A. M.. (2016). The oral microbiome - an update for oral healthcare professionals. Br. Dent. J. 221, 657–666. doi: 10.1038/sj.bdj.2016.865 27857087

[B65] Kim S. S. Ruiz V. E. Carroll J. D. Moss S. F. (2011). Helicobacter pylori in the pathogenesis of gastric cancer and gastric lymphoma. Cancer Letters. 305, 228–238. doi: 10.1016/j.canlet.2010.07.014 20692762 PMC2980557

[B66] Kobayashi J. Ohtake K. Uchida H. (2015). NO-rich diet for lifestyle-related diseases. Nutrients. 7, 4911–4937. doi: 10.3390/nu7064911 26091235 PMC4488823

[B67] Korde Choudhari S. Chaudhary M. Bagde S. Gadbail A. R. Joshi V. (2013). Nitric oxide and cancer: a review. World J. Surg. Oncol. 11, 1–11. doi: 10.1186/1477-7819-11-118 23718886 PMC3669621

[B68] Kozak M. Pawlik A. (2023). The role of the oral microbiome in the development of diseases. Int. J. Mol. Sci. 24, 5231. doi: 10.3390/ijms24065231 36982305 PMC10048844

[B69] Krasse B. (2001). The Vipeholm Dental Caries Study: recollections and reflections 50 years later. J. Dental Res. 80, 1785–1788. doi: 10.1177/00220345010800090201 11926233

[B70] Król M. Kepinska M. (2020). Human nitric oxide Synthase—Its functions, polymorphisms, and inhibitors in the context of inflammation, diabetes and cardiovascular diseases. Int. J. Mol. Sci. 22, 56. doi: 10.3390/ijms22010056 33374571 PMC7793075

[B71] Lamont R. J. Hajishengallis G. (2015). Polymicrobial synergy and dysbiosis in inflammatory disease. Trends Mol. Med. 21, 172–183. doi: 10.1016/j.molmed.2014.11.004 25498392 PMC4352384

[B72] Li Q. Lan Q. Zhang Y. Bassig B. A. Holford T. R. Leaderer B.. (2013). Role of one-carbon metabolizing pathway genes and gene-nutrient interaction in the risk of non-Hodgkin lymphoma. Cancer Causes Control. 24, 1875–1884. doi: 10.1007/s10552-013-0264-3 23913011 PMC3951097

[B73] Li B.-Z. Zhou H.-Y. Guo B. Chen W.-J. Tao J.-H. Cao N.-W.. (2020). Dysbiosis of oral microbiota is associated with systemic lupus erythematosus. Arch. Oral. Biol. 113, 104708. doi: 10.1016/j.archoralbio.2020.104708 32203722

[B74] Lin S. Li Y. Zamyatnin A. A. Jr. Werner J. Bazhin A. V. (2018). Reactive oxygen species and colorectal cancer. J. Cell. Physiol. 233, 5119–5132. doi: 10.1002/jcp.v233.7 29215746

[B75] Liu Y. Croft K. D. Hodgson J. M. Mori T. Ward N. C. (2020). Mechanisms of the protective effects of nitrate and nitrite in cardiovascular and metabolic diseases. Nitric. Oxide 96, 35–43. doi: 10.1016/j.niox.2020.01.006 31954804

[B76] Liu J. Li K. Dou K. (2003). The Expression of iNOS and COX-2 in Pancreatic Cancer and its Clinical Significance. Cancer Res. Prev. Treat. 30, 361. doi: 10.3971/j.issn.1000-8578.1426

[B77] Lloyd-Price J. Abu-Ali G. Huttenhower C. (2016). The healthy human microbiome. Genome Med. 8, 51. doi: 10.1186/s13073-016-0307-y 27122046 PMC4848870

[B78] Luetic S. Knezovic Z. Jurcic K. Majic Z. Tripkovic K. Sutlovic D. (2023). Leafy vegetable nitrite and nitrate content: potential health effects. Foods. 12, 45–62. doi: 10.3390/foods12081655 PMC10137473 37107450

[B79] Luetic S. Knezovic Z. Jurcic K. Perasovic M. L. Sutlovic D. (2025). Nitrates and nitrites in leafy vegeta bles: the influence of culinary processing on concentration levels and possible impact on health. Int. J. Mol. Sci. 26, 3018. doi: 10.3390/ijms26073018 40243642 PMC11988860

[B80] Lundberg J. O. Weitzberg E. (2022). Nitric oxide signaling in health and disease. Cell. 185, 2853–2878. doi: 10.1016/j.cell.2022.06.010 35931019

[B81] Lundberg J. O. Weitzberg E. Gladwin M. T. (2008a). The nitrate-nitrite-nitric oxide pathway in physiology and therapeutics. Nat. Rev. Drug Discov. 7, 156–167. doi: 10.1038/nrd2466 18167491

[B82] Lundberg J. O. Weitzberg E. Gladwin M. T. (2008b). The nitrate–nitrite–nitric oxide pathway in physiology and therapeutics. Nat. Rev. Drug discovery. 7, 156–167. doi: 10.1038/nrd2466 18167491

[B83] Ma J. Xia J. Miele L. Sarkar F. H. Wang Z. (2013). Notch signaling pathway in pancreatic cancer progression. Pancreat Disord. Ther. 3, 101–115. doi: 10.4172/2165-7092.1000114 PMC3767173 24027656

[B84] Ma L. F. Xu L. L. Yuan L. J. Yang X. Wu R. Bao S. M.. (2024). Discovery of NO donor-aurovertin hybrids as dual ferroptosis and apoptosis inducers for treating triple negative breast cancer. J. Med. Chem. 67, 13089–13105. doi: 10.1021/acs.jmedchem.4c01070 39044437

[B85] Mazurek M. Rola R. (2021). The implications of nitric oxide metabolism in the treatment of glial tumors. Neurochemistry Int. 150, 105172. doi: 10.1016/j.neuint.2021.105172 34461111

[B86] McBee M. E. Chionh Y. H. Sharaf M. L. Ho P. Cai M. W. L. Dedon P. C. (2017). Production of superoxide in bacteria is stress- and cell state-dependent: A gating-optimized flow cytometry method that minimizes ROS measurement artifacts with fluorescent dyes. Front. Microbiol. 459 2017 doi: 10.3389/fmicb.2017.00459 PMC5359317 28377755

[B87] McCurdy M. R. Wazni M. W. Martinez J. McAleer M. F. Guerrero T. (2011). Exhaled nitric oxide predicts radiation pneumonitis in esophageal and lung cancer patients receiving thoracic radiation. Radiotherapy Oncol. 101, 443–448. doi: 10.1016/j.radonc.2011.08.035 21981878

[B88] Mehta A. Patel B. M. (2019). Therapeutic opportunities in colon cancer: Focus on phosphodiesterase inhibitors. Life Sci. 230, 150–161. doi: 10.1016/j.lfs.2019.05.043 31125564

[B89] Merenzon M. A. Hincapie Arias E. Bhatia S. Shah A. H. Higgins D. M. O. Villaverde M.. (2023). Nitric oxide synthase inhibitors as potential therapeutic agents for gliomas: A systematic review. Nitric. Oxide 138-139, 10–16. doi: 10.1016/j.niox.2023.06.002 37279819

[B90] Mintz J. Vedenko A. Rosete O. Shah K. Goldstein G. Hare J. M.. (2021). Current advances of nitric oxide in cancer and anticancer therapeutics. Vaccines. 9, 94. doi: 10.3390/vaccines9020094 33513777 PMC7912608

[B91] Mokry R. L. Schumacher M. L. Hogg N. Terhune S. S. (2020). Nitric oxide circumvents virus-mediated metabolic regulation during human cytomegalovirus infection. Mbio. 11, 230–245. doi: 10.1128/mbio.02630-20 PMC7773989 33323506

[B92] Monteiro H. Costa P. Reis A. Stern A. (2015). Nitric oxide: protein tyrosine phosphorylation and protein S-nitrosylation in cancer. Biomed. J. 38, 88–95. doi: 10.4103/2319-4170.158624 26068128

[B93] Monteiro H. P. Rodrigues E. G. Amorim Reis A. K. C. Longo L. S. Jr. Ogata F. T. Moretti A. I. S.. (2019). Nitric oxide and interactions with reactive oxygen species in the development of melanoma, breast, and colon cancer: A redox signaling perspective. Nitric. Oxide 89, 1–13. doi: 10.1016/j.niox.2019.04.009 31009708

[B94] Moran S. P. Rosier B. T. Henriquez F. L. Burleigh M. C. (2024). The effects of nitrate on the oral microbiome: a systematic review investigating prebiotic potential. J. Oral. Microbiol. 16, 2322228. doi: 10.1080/20002297.2024.2322228 38420038 PMC10901185

[B95] Morou-Bermúdez E. Torres-Colón J. E. Bermúdez N. S. Patel R. P. Joshipura K. J. (2022). Pathways linking oral bacteria, nitric oxide metabolism, and health. J. Dental Res. 101, 623–631. doi: 10.1177/00220345211064571 PMC9124908 35081826

[B96] Morrison A. G. Sarkar S. Umar S. Lee S. T. Thomas S. M. (2023). The contribution of the human oral microbiome to oral disease: A review. Microorganisms. 11, 318. doi: 10.3390/microorganisms11020318 36838283 PMC9962706

[B97] Mubayi A. Greenwood P. Wang X. Castillo-Chávez C. Gorman D. M. Gruenewald P.. (2011). Types of drinkers and drinking settings: an application of a mathematical model. Addiction. 106, 749–758. doi: 10.1111/j.1360-0443.2010.03254.x 21182556 PMC3057526

[B98] Nafea H. Youness R. A. Abou-Aisha K. Gad M. Z. (2021). LncRNA HEIH/miR-939-5p interplay modulates triple-negative breast cancer progression through NOS2-induced nitric oxide production. J. Cell Physiol. 236, 5362–5372. doi: 10.1002/jcp.v236.7 33368266

[B99] Narikiyo M. Tanabe C. Yamada Y. Igaki H. Tachimori Y. Kato H.. (2004). Frequent and preferential infection of Treponema denticola, Streptococcus mitis, and Streptococcus anginosus in esophageal cancers. Cancer science. 95, 569–574. doi: 10.1111/j.1349-7006.2004.tb02488.x 15245592 PMC11159681

[B100] Ndegwa N. Ploner A. Liu Z. Roosaar A. Axéll T. Ye W. (2018). Association between poor oral health and gastric cancer: a prospective cohort study. Int. J. Cancer. 143, 2281–2288. doi: 10.1002/ijc.v143.9 29873081

[B101] Niu X.-J. Wang Z.-R. Wu S.-L. Geng Z.-M. Zhang Y.-F. Qing X.-L. (2004). Relationship between inducible nitric oxide synthase expression and angiogenesis in primary gallbladder carcinoma tissue. World J. gastroenterology. 10, 725. doi: 10.3748/wjg.v10.i5.725 PMC4716918 14991947

[B102] Obidiro O. Battogtokh G. Akala E. O. (2023). Triple negative breast cancer treatment options and limitations: future outlook. Pharmaceutics. 15, 312–328. doi: 10.3390/pharmaceutics15071796 37513983 PMC10384267

[B103] Olas B. (2024). The cardioprotective role of nitrate-rich vegetab les. Foods. 13, 691. doi: 10.3390/foods13050691 38472804 PMC10931520

[B104] Pacher P. Beckman J. S. Liaudet L. (2007). Nitric oxide and peroxynitrite in health and disease. Physiol. Rev. 87, 315–424. doi: 10.1152/physrev.00029.2006 17237348 PMC2248324

[B105] Park J.-E. Seo J.-E. Lee J.-Y. Kwon H. (2015). Distribution of seven N-nitrosamines in food. Toxicological Res. 31, 279–288. doi: 10.5487/TR.2015.31.3.279 PMC4609975 26483887

[B106] PeÑarando J. Aranda E. RodrÍguez-Ariza A. (2019). Immunomodulatory roles of nitric oxide in cancer: tumor microenvironment says “NO” to antitumor immune response. Transl. Res. 210, 99–108. doi: 10.1016/j.trsl.2019.03.003 30953610

[B107] Pennisi M. Lanza G. Cantone M. Schepis C. Ferri R. Barone R.. (2017). Unusual neurological presentation of nevoid basal cell carcinoma syndrome (Gorlin-Goltz syndrome). J. Clin. Neurology. 13, 439–441. doi: 10.3988/jcn.2017.13.4.439 PMC5653638 28884983

[B108] Pignatelli P. Iezzi L. Pennese M. Raimondi P. Cichella A. Bondi D.. (2021). The potential of colonic tumor tissue fusobacterium nucleatum to predict staging and its interplay with oral abundance in colon cancer patients. Cancers (Basel) 13, 175–190. doi: 10.3390/cancers13051032 33804585 PMC7957509

[B109] Pinheiro L. C. Amaral J. H. Ferreira G. C. Portella R. L. Ceron C. S. Montenegro M. F.. (2015). Gastric S-nitrosothiol formation drives the antihypertensive effects of oral sodium nitrite and nitrate in a rat model of renovascular hypertension. Free Radical Biol. Med. 87, 252–262. doi: 10.1016/j.freeradbiomed.2015.06.038 26159506

[B110] Plenchette S. Paul C. Bettaieb A. (2017). “Chapter 6 - nitric oxide and platinum-derivative-based regimens for cancer treatment: from preclinical studies to clinical trials,” in Nitric oxide (Donor/induced) in chemosensitizing. 1. Ed. Bonavida B. (Elsevier, Amsterdam, Netherlands: Academic Press), 91–103.

[B111] Radi R. (2013). Peroxynitrite, a stealthy biological oxidant. J. Biol. Chem. 288, 26464–26472. doi: 10.1074/jbc.R113.472936 23861390 PMC3772193

[B112] Rajnakova A. Moochhala S. Goh P. M. Ngoi S.-S. (2001). Expression of nitric oxide synthase, cyclooxygenase, and p53 in different stages of human gastric cancer. Cancer letters. 172, 177–185. doi: 10.1016/S0304-3835(01)00645-0 11566494

[B113] Rosier B. T. Marsh P. D. Mira A. (2018). Resilience of the oral microbiota in health: mechanisms that prevent dysbiosis. J. Dent. Res. 97, 371–380. doi: 10.1177/0022034517742139 29195050

[B114] Rubinstein M. R. Wang X. Liu W. Hao Y. Cai G. Han Y. W. (2013). Fusobacterium nucleatum promotes colorectal carcinogenesis by modulating E-cadherin/β-catenin signaling via its FadA adhesin. Cell Host Microbe 14, 195–206. doi: 10.1016/j.chom.2013.07.012 23954158 PMC3770529

[B115] Saaed H. K. Chiggiato L. Webb D.-L. Rehnberg A.-S. Rubio C. A. Befrits R.. (2021). Elevated gaseous luminal nitric oxide and circulating IL-8 as features of Helicobacter pylori-induced gastric inflammation. Upsala J. Med. Sci. 126, 50–67. doi: 10.48101/ujms.v126.8116 PMC8559587 34754406

[B116] Said Abasse K. Essien E. E. Abbas M. Yu X. Xie W. Sun J.. (2022). Association between dietary nitrate, nitrite intake, and site-specific cancer risk: A systematic review and meta-analysis. Nutrients 14, 120–135. doi: 10.3390/nu14030666 PMC8838348 35277025

[B117] Salvatori L. Malatesta S. Illi B. Somma M. P. Fionda C. Stabile H.. (2023). Nitric oxide prevents glioblastoma stem cells’ Expansion and induces temozolomide sensitization. Int. J. Mol. Sci. 24, 11286. doi: 10.3390/ijms241411286 37511047 PMC10379318

[B118] Sang J. Chen Y. Tao Y. (2011). Nitric oxide inhibits gastric cancer cell growth through the modulation of the Akt pathway. Mol. Med. Rep. 4, 1163–1167. doi: 10.3892/mmr.2011.535 21769431

[B119] Sarkar P. Malik S. Laha S. Das S. Bunk S. Ray J. G.. (2021). Dysbiosis of oral microbiota during oral squamous cell carcinoma development. Front. Oncol. 11, 614448. doi: 10.3389/fonc.2021.614448 33708627 PMC7940518

[B120] Sasaki H. Ishizuka T. Muto M. Nezu M. Nakanishi Y. Inagaki Y.. (1998). Presence of Streptococcus anginosus DNA in esophageal cancer, dysplasia of esophagus, and gastric cancer. Cancer Res. 58, 2991–2995. 9679961

[B121] Sayır F. Şehitoğulları A. Demir H. Aslan M. Çobanoğlu U. Bilgin C. (2019). Serum prolidase activity, total oxidant/antioxidant, and nitric oxide levels in patients with esophageal squamous cell carcinoma. Turkish J. Thorac. Cardiovasc. Surgery. 27, 206. doi: 10.5606/tgkdc.dergisi.2019.16888 PMC7021385 32082854

[B122] Schenk M. W. Humphrey S. Hossain A. Revill M. Pearsall S. Lallo A.. (2021). Soluble guanylate cyclase signalling mediates etoposide resistance in progressing small cell lung cancer. Nat. Commun. 12, 6652. doi: 10.1038/s41467-021-26823-6 34789728 PMC8599617

[B123] Schullehner J. Hansen B. Thygesen M. Pedersen C. B. Sigsgaard T. (2018). Nitrate in drinking water and colorectal cancer risk: A nationwide population-based cohort study. Int. J. cancer. 143, 73–79. doi: 10.1002/ijc.v143.1 29435982

[B124] Schwartz M. Canon F. Feron G. Neiers F. Gamero A. (2021). Impact of oral microbiota on flavor perception: From food processing to in-mouth metabolization. Foods. 10, 2006. doi: 10.3390/foods10092006 34574116 PMC8467474

[B125] Semenikhina M. Stefanenko M. Spires D. R. Ilatovskaya D. V. Palygin O. (2022). Nitric-oxide-mediated signaling in podocyte pathophysiology. Biomolecules. 12, 200–216. doi: 10.3390/biom12060745 35740870 PMC9221338

[B126] Sharma T. Gupta A. Chauhan R. Bhat A. A. Nisar S. Hashem S.. (2022). Cross-talk between the microbiome and chronic inflammation in esophageal cancer: potential driver of oncogenesis. Cancer Metastasis Rev. 41, 281–299. doi: 10.1007/s10555-022-10026-6 35511379 PMC9363391

[B127] Shi N. Chen T. (2022). Chemopreventive properties of black raspberries and strawberries in esophageal cancer review. Antioxidants. 11, 1815. doi: 10.3390/antiox11091815 36139889 PMC9495642

[B128] Shiva S. (2013). Nitrite: A physiological store of nitric oxide and modulator of mitochondrial function. Redox Biol. 1, 40–44. doi: 10.1016/j.redox.2012.11.005 23710434 PMC3661298

[B129] Shouval R. Eshel A. Dubovski B. Kuperman A. A. Danylesko I. Fein J. A.. (2020). Patterns of salivary microbiota injury and oral mucositis in recipients of allogeneic hematopoietic stem cell transplantation. Blood advances. 4, 2912–2917. doi: 10.1182/bloodadvances.2020001827 32598476 PMC7362373

[B130] Siegel R. L. Miller K. D. Goding Sauer A. Fedewa S. A. Butterly L. F. Anderson J. C.. (2020). Colorectal cancer statistics, 2020. CA Cancer J. Clin. 70, 145–164. doi: 10.3322/caac.21601 32133645

[B131] Somasundaram V. Ridnour L. A. Cheng R. Y. S. Walke A. J. Kedei N. Bhattacharyya D. D.. (2022). Systemic Nos2 Depletion and Cox inhibition limits TNBC disease progression and alters lymphoid cell spatial orientation and density. Redox Biol. 58, 102529. doi: 10.1016/j.redox.2022.102529 36375380 PMC9661390

[B132] Song P. Wu L. Guan W. (2015). Dietary nitrates, nitrites, and nitrosamines intake and the risk of gastric cancer: A meta-analysis. Nutrients. 7, 9872–9895. doi: 10.3390/nu7125505 26633477 PMC4690057

[B133] Srour B. Chazelas E. Druesne-Pecollo N. Esseddik Y. de Edelenyi F. S. Agaësse C.. (2023). Dietary exposure to nitrites and nitrates in association with type 2 diabetes risk: Results from the NutriNet-Santé population-based cohort study. PloS Med. 20, e1004149. doi: 10.1371/journal.pmed.1004149 36649248 PMC9844911

[B134] Su H. Liu X. Du J. Deng X. Fan Y. (2020). The role of hemoglobin in nitric oxide transport in vascular system. Med. Novel Technol. Devices. 5, 100034. doi: 10.1016/j.medntd.2020.100034

[B135] Suksawat M. Techasen A. Namwat N. Yongvanit P. Khuntikeo N. Titapun A.. (2017). Upregulation of endothelial nitric oxide synthase (eNOS) and its upstream regulators in Opisthorchis viverrini associated cholangiocarcinoma and its clinical significance. Parasitol. Int. 66, 486–493. doi: 10.1016/j.parint.2016.04.008 27143607

[B136] Sun G.-G. Hu W.-N. Zhang J. Li C.-L. Yang C.-R. (2013). Effect of nitric oxide on esophageal cancer cell line TE-1. Chin. Med. Sci. J. 28, 44–49. doi: 10.1016/S1001-9294(13)60018-8 23527807

[B137] Sun J. Tang Q. Yu S. Xie M. Xie Y. Chen G.. (2020). Role of the oral microbiota in cancer evolution and progression. Cancer Med. 9, 6306–6321. doi: 10.1002/cam4.v9.17 32638533 PMC7476822

[B138] Sun J. Zhou M. Salazar C. R. Hays R. Bedi S. Chen Y.. (2017). Chronic periodontal disease, periodontal pathogen colonization, and increased risk of precancerous gastric lesions. J. Periodontol. 88, 1124–1134. doi: 10.1902/jop.2017.160829 28671506

[B139] Sung H. Ferlay J. Siegel R. L. Laversanne M. Soerjomataram I. Jemal A.. (2021). Global cancer statistics 2020: GLOBOCAN estimates of incidence and mortality worldwide for 36 cancers in 185 countries. CA Cancer J. Clin. 71, 209–249. doi: 10.3322/caac.21660 33538338

[B140] Tanda N. Washio J. Kamei T. Akazawa K. Takahashi N. Koseki T. (2019). Professional oral care reduces carcinogenic acetaldehyde levels in mouth air of perioperative esophageal cancer patients: A prospective comparative study. Tohoku J. Exp. Med. 249, 75–83. doi: 10.1620/tjem.249.75 31564686

[B141] Tandon P. Dadhich A. Saluja H. Bawane S. Sachdeva S. (2017). The prevalence of squamous cell carcinoma in different sites of oral cavity at our Rural Health Care Centre in Loni, Maharashtra–a retrospective 10-year study. Contemp. Oncology/Współczesna Onkologia. 21, 178–183. doi: 10.5114/wo.2017.68628 PMC5611509 28947890

[B142] Tateda M. Shiga K. Saijo S. Sone M. Hori T. Yokoyama J.. (2000). Streptococcus anginosus in head and neck squamous cell carcinoma: implication in carcinogenesis. Int. J. Mol. Med. 6, 699–1402. doi: 10.3892/ijmm.6.6.699 11078831

[B143] Temkin A. Evans S. Manidis T. Campbell C. Naidenko O. V. (2019). Exposure-based assessment and economic valuation of adverse birth outcomes and cancer risk due to nitrate in United States drinking water. Environ. Res. 176, 108442. doi: 10.1016/j.envres.2019.04.009 31196558

[B144] Thaiss C. A. Zmora N. Levy M. Elinav E. (2016). The microbiome and innate immunity. Nature. 535, 65–74. doi: 10.1038/nature18847 27383981

[B145] Tiso M. Schechter A. N. (2015). Nitrate reduction to nitrite, nitric oxide and ammonia by gut bacteria under physiological conditions. PloS One 10, e0119712. doi: 10.1371/journal.pone.0119712 25803049 PMC4372352

[B146] Tripathi M. K. Ojha S. K. Amal H. (2023). 532P Nitric oxide is a target by a combo-drug for glioblastoma treatment. Ann. Oncol. 34, S403. doi: 10.1016/j.annonc.2023.09.1726

[B147] Trombelli L. Tatakis D. N. Scapoli C. Bottega S. Orlandini E. Tosi M. (2004). Modulation of clinical expression of plaque-induced gingivitis: II. Identification of “high-responder” and “low-responder” subjects. J. Clin. periodontology. 31, 239–252. doi: 10.1111/j.1600-051x.2004.00478.x 15016251

[B148] Tuominen H. Rautava J. (2021). Oral microbiota and cancer development. Pathobiology. 88, 116–126. doi: 10.1159/000510979 33176328

[B149] Uchino Y. Goto Y. Konishi Y. Tanabe K. Toda H. Wada M.. (2021). Colorectal cancer patients have four specific bacterial species in oral and gut microbiota in common-A metagenomic comparison with healthy subjects. Cancers (Basel) 13, 300–315. doi: 10.3390/cancers13133332 34283063 PMC8268706

[B150] Uhlenhopp D. J. Then E. O. Sunkara T. Gaduputi V. (2020). Epidemiology of esophageal cancer: update in global trends, etiology and risk factors. Clin. J. Gastroenterol. 13, 1010–1021. doi: 10.1007/s12328-020-01237-x 32965635

[B151] Voss N. C. Kold-Petersen H. Boedtkjer E. (2019). Enhanced nitric oxide signaling amplifies vasorelaxation of human colon cancer feed arteries. Am. J. Physiology-Heart Circulatory Physiol. 316, H245–HH54. doi: 10.1152/ajpheart.00368.2018 30444664

[B152] Wade W. G. (2013). The oral microbiome in health and disease. Pharmacol. Res. 69, 137–143. doi: 10.1016/j.phrs.2012.11.006 23201354

[B153] Wade W. G. (2021). Resilience of the oral microbiome. Periodontology 2000. 86, 113–122. doi: 10.1111/prd.12365 33690989

[B154] Wang J. He P. Gaida M. Yang S. Schetter A. J. Gaedcke J.. (2016a). Inducible nitric oxide synthase enhances disease aggressiveness in pancreatic cancer. Oncotarget. 7, 52993. doi: 10.18632/oncotarget.10323 27367029 PMC5288163

[B155] Wang J. He P. Gaida M. Yang S. Schetter A. J. Gaedcke J.. (2016b). Inducible nitric oxide synthase enhances disease aggressiveness in pancreatic cancer. Oncotarget. 7, 52993–53004. doi: 10.18632/oncotarget.10323 27367029 PMC5288163

[B156] Wang H. Wang L. Xie Z. Zhou S. Li Y. Zhou Y.. (2020). Nitric oxide (NO) and NO synthases (NOS)-based targeted therapy for colon cancer. Cancers. 12, 1881. doi: 10.3390/cancers12071881 32668616 PMC7408898

[B157] Wang Q. Ye S. Chen X. Xu P. Li K. Zeng S.. (2019). Mitochondrial NOS1 suppresses apoptosis in colon cancer cells through increasing SIRT3 activity. Biochem. Biophys. Res. Commun. 515, 517–523. doi: 10.1016/j.bbrc.2019.05.114 31153640

[B158] Ward M. H. Jones R. R. Brender J. D. de Kok T. M. Weyer P. J. Nolan B. T.. (2018). Drinking water nitrate and human health: an updated review. Int. J. Environ. Res. Public Health 15, 80–95. doi: 10.3390/ijerph15071557 30041450 PMC6068531

[B159] Welch J. L. M. Ramírez-Puebla S. T. Borisy G. G. (2020). Oral microbiome geography: micron-scale habitat and niche. Cell Host Microbe 28, 160–168. doi: 10.1016/j.chom.2020.07.009 32791109 PMC7604680

[B160] Wink D. A. Hines H. B. Cheng R. Y. Switzer C. H. Flores-Santana W. Vitek M. P.. (2011). Nitric oxide and redox mechanisms in the immune response. J. Leukoc. Biol. 89, 873–891. doi: 10.1189/jlb.1010550 21233414 PMC3100761

[B161] Xie L. Mo M. Jia H.-X. Liang F. Yuan J. Zhu J. (2016). Association between dietary nitrate and nitrite intake and site-specific cancer risk: evidence from observational studies. Oncotarget. 7, 150–165. doi: 10.18632/oncotarget.10917 PMC5302962 27486968

[B162] Xiong H. Wang R. Zhang C. (2021). A review on the research progress of inducible nitric oxide synthase in the pathogenesis of pancreatic Malignancy. J. Clin. Nurs. Res. 5, 151–153. doi: 10.26689/jcnr.v5i4.2281

[B163] Yang Y. Long J. Wang C. Blot W. J. Pei Z. Shu X.. (2022). Prospective study of oral microbiome and gastric cancer risk among Asian, African American and European American populations. Int. J. Cancer. 150, 916–927. doi: 10.1002/ijc.v150.6 34664266 PMC8982516

[B164] Yao X. Wu Y. Zhu M. Qian H. Chen Y. (2015). Nitric oxide/cyclic guanosine monophosphate inducers sodium nitroprusside and L-arginine inhibit the proliferation of gastric cancer cells via the activation of type II cyclic guanosine monophosphate-dependent protein kinase. Oncol. Letters. 10, 479–484. doi: 10.3892/ol.2015.3229 PMC4487134 26171055

[B165] Zhang X. Jin L. Tian Z. Wang J. Yang Y. Liu J.. (2019). Nitric oxide inhibits autophagy and promotes apoptosis in hepatocellular carcinoma. Cancer science. 110, 1054–1063. doi: 10.1111/cas.2019.110.issue-3 30657629 PMC6398894

[B166] Zhang L. Liu Y. Zheng H. J. Zhang C. P. (2019). The oral microbiota may have influence on oral cancer. Front. Cell Infect. Microbiol. 9, 476. doi: 10.3389/fcimb.2019.00476 32010645 PMC6974454

[B167] Zhang Y. Wang X. Li H. Ni C. Du Z. Yan F. (2018). Human oral microbiota and its modulation for oral health. Biomedicine Pharmacotherapy. 99, 883–893. doi: 10.1016/j.biopha.2018.01.146 29710488

[B168] Zhang W.-L. Wang S.-S. Wang H.-F. Tang Y.-J. Tang Y.-L. Liang X.-H. (2019). Who is who in oral cancer? Exp. Cell Res. 384, 111634. doi: 10.1016/j.yexcr.2019.111634 31541617

[B169] Zhang Y. Z. Wang C. F. Zhang L. F. (2018). Cucurbitacin D impedes gastric cancer cell survival via activation of the iNOS/NO and inhibition of the Akt signalling pathway. Oncol. Rep. 39, 2595–2603. doi: 10.3892/or.2018.6361 29658590 PMC5983931

[B170] Zhao J. O’Neil M. Schonfeld M. Komatz A. Weinman S. A. Tikhanovich I. (2020). Hepatocellular protein arginine methyltransferase 1 suppresses alcohol-induced hepatocellular carcinoma formation by inhibition of inducible nitric oxide synthase. Hepatol. Commun. 4, 790–808. doi: 10.1002/hep4.1488 32490317 PMC7262284

[B171] Zhou L. Wang Y. Tian D.-A. Yang J. Yang Y.-Z. (2012). Decreased levels of nitric oxide production and nitric oxide synthase-2 expression are associated with the development and metastasis of hepatocellular carcinoma. Mol. Med. Rep. 6, 1261–1266. doi: 10.3892/mmr.2012.1096 23007408

[B172] Zhou L. Zhang H. Wu J. (2016). Effects of nitric oxide on the biological behavior of HepG2 human hepatocellular carcinoma cells. Exp. Ther. Med. 11, 1875–1880. doi: 10.3892/etm.2016.3128 27168820 PMC4840525

[B173] Zou D. Li Z. Lv F. Yang Y. Yang C. Song J.. (2021). Pan-cancer analysis of NOS3 identifies its expression and clinical relevance in gastric cancer. Front. Oncol. 11, 592761. doi: 10.3389/fonc.2021.592761 33747912 PMC7969995

